# Quantitative Indices of Student Social Media Engagement in Tertiary Education: A Systematic Review and a Taxonomy

**DOI:** 10.1007/s10864-023-09516-6

**Published:** 2023-04-12

**Authors:** Aida Tarifa-Rodriguez, Javier Virues-Ortega, Agustin Perez-Bustamante Pereira, Ana Calero-Elvira, Sarah Cowie

**Affiliations:** 1 Universidad Autónoma de Madrid, Madrid, Spain; 2 The University of Auckland, Auckland, New Zealand; 3 Hospital Universitario Ramón y Cajal, Madrid, Spain

**Keywords:** Social media, Social media engagement, Achievement, Tertiary education, Behavioral engagement, Online education

## Abstract

Recent studies have evaluated the use of social media as learning aids in tertiary education. Emerging research in this area has focused primarily on non-quantitative approaches to student social media engagement. However, quantitative engagement outcomes may be extracted from student posts, comments, likes, and views. The goal of the present review was to provide a research-informed taxonomy of quantitative and behavior-based metrics of student social media engagement. We selected 75 empirical studies comprising a pooled sample of 11,605 tertiary education students. Included studies used social media for educational purposes and reported student social media engagement outcomes (source databases: PsycInfo and ERIC). We used independent raters and stringent interrater agreement and data extraction processes to mitigate bias during the screening of references. Over half of the studies (52%, *n* = 39) utilized ad hoc interviews and surveys to estimate student social media engagement, whereas thirty-three studies (44%) used some form of quantitative analysis of engagement. Based on this literature, we present a selection of count-based, time-based, and text-analysis metrics. The proposed taxonomy of engagement metrics resulting provides the methodological basis for the analysis of social media behavior in educational settings, particularly, for human operant and behavioral education studies. Implications for future research are discussed.

**Supplementary Information:**

The online version contains supplementary material available at 10.1007/s10864-023-09516-6.

Social media have been defined as Internet applications that allow users to connect and interact with each other while creating, sharing, or reacting to online content (see, for example, Kaplan & Haenlein, 2010). From a behavioral standpoint, social media is a complex social environment where users engage in a diverse range of online behaviors receiving a variety of social and automated consequences as a result. It is expected that social media platforms will reach five billion users by 2024, with platforms such as Facebook, Instagram, WhatsApp, YouTube, TikTok, and Twitter serving as the main communication networks (Statista, 2021, 2023). This trend is particularly prominent among university students. For example, a recent survey indicated that 82% of tertiary education students are regular Facebook users (Statista, 2022). The global COVID-19 pandemic galvanized an on-going shift towards online education, expediting the adoption of teaching strategies, content design standards, and time management processes aligned with the new medium (Papademetriou et al., 2022). Some institutions quickly enabled educational platforms (e-learning platforms), while those with less resources relied on social media platforms as a readily available educational channel (Sobaih et al., 2022).

Despite these trends, universities have only just started to use these platforms for educational purposes. Social media has the potential of supporting traditional classroom environments by adding accessible, barrier-free virtual spaces that could enhance collaborative peer- and instructor-mediated learning. There is evidence to suggest that student engagement in social media discussions moderated by an instructor may be an important indicator of course content elaboration and social learning (Parks-Stamm et al., 2017).

Studies within this emerging field often rely on indirect measures of engagement (e.g., satisfaction surveys, teacher reports), which fail to describe the quantitative dimensions of online behavior; dimensions such as frequency, latency, and intensity (Giannikas, 2019; Slim & Hafedh, 2019). Social media platforms make it possible to quantify student engagement in a variety of ways. For example, most social media platforms log the exact time and date of posts, comments, and user reactions (e.g., likes) affording a myriad of metrics (e.g., posting frequency, comment latency). These metrics have the potential to inform the teaching–learning process when social medial channels are used in educational contexts.

Quantitative measures of engagement allow us not only to quantify the effectiveness of interventions directed at increasing engagement, but also to detect operant learning mechanisms such as reinforcement, extinction, and punishment, that could be influencing students’ online behavior (Honig & Staddon, 2022). Recently, Lindström et al. (2021) used a computational approach to assess whether operant processes could explain engagement responses in social media. These authors used response "latency" (the time elapsed between two successive social media posts) as an indicator of engagement. Their results showed that users of social media platforms space out their posts according to a model of social reinforcement maximization. This finding may have implications for the use of social media for educational purposes.

An operant model of social media interaction could provide the conceptual basis for future evidence-based strategies to foster positive and learning-enhancing interventions, for example, by using online social rewards such as offering immediate or near-immediate feedback. Moreover, it may be possible to create educational contexts in which high rates of social reinforcement are available for appropriate engagement, which could ultimately maximize academic performance. We could also obtain evidence of operant behavior allocation by monitoring posting behavior at times when instructor responses have a shorter latency or are more relevant (e.g., specific feedback), relative to times when instructor responses are delayed or are less relevant (e.g., collective feedback). More frequent posting in the former scenario and less frequent posting in the latter would provide evidence (whether correlational or experimental) of operant behavior allocation. The analyses suggested above may have direct practical implications. In order to evaluate operant processes in the social media context, it would be necessary to establish quantitative metrics of discrete student and instructor social media responses.

While the literature on the use of social media for educational purposes has grown steadily over the last decade (Tawafak et al., 2021), most of this research seems to be qualitative and does not contain behavioral data on engagement or performance (Papademetriou et al., 2022), making it difficult to capitalize on this important line of research. In addition, in order to evaluate an operant model of social media interaction in educational settings, it would be important to define and validate behaviorally based quantitative metrics of social media interaction. For example, Lindström et al. (2021) were able to demonstrate reinforcement effects by focusing on inter-post time (which they labeled "latency") as a key social media engagement metric. Further progress in this area in both human-operant and applied studies would require a better understanding of the quantitative metrics that can be retrieved from the social dynamics in this medium. While numerous classifications of generalist behavioral metrics have been published over the years (e.g., Floyd et al., 1998; Johnston et al., 2019) and there are a few systematic reviews on the general theme of online behavior in social media (see for example Masrom et al., 2021), we are not aware of any systematic reviews of behavior-based metrics that could be obtained from social media platforms.

The Linnaean and Mendeleev systems have been instrumental to the development of the evolutionary and atomic theories (see, for example, Hettema & Kuipers, 1988, and Paterlini, 2007). Likewise, the role of methodological taxonomies has been amply recognized in psychology as a preliminary step for conceptual development and applied research (see for example Chafetz, 1986). Similarly, functional taxonomies in behavior analysis have played a key role in galvanizing conceptual, technological, and applied advances. For example, the classification of problem behavior function by the stimulus dimension of the reinforcer (social vs. automatic) and the stimulus manipulation preceding changes in behavior (positive vs. negative reinforcement), led to the development of functional analysis methodology by Iwata et al. (1982), which in turn has resulted in further refinements in functional analysis outcomes subtypes (see, for example, Virues-Ortega et al., 2022a, 2022b). In this connection, the development of a behavior-analytic research subfield of social media online behavior would greatly benefit from a systematic classification of behavior metrics and dimensions that could be utilized to study the interactions of individuals with online platforms and with other users in the medium.

The goal of the current study was to review the literature that has evaluated social media engagement in the context of tertiary education programs with integrated social media platforms to determine the relative presence of qualitative and quantitative engagement outcomes. This evidence will be used to ascertain the basic trends in this literature and as the basis for a preliminary taxonomy of quantitative engagement metrics that could be widely used in human-operant research and applied behavioral education.

## Methods

### Study Selection

We conducted a comprehensive literature search in the PsycInfo and ERIC databases (ProQuest search engine) on October 28, 2020. After repeated preliminary searchers to test search sensitivity, the following search strategy was implemented: (“Facebook” OR “social media”) AND (“engagement,” “education,” OR “achievement”) without time or search field restrictions.

**Fig. 1:**
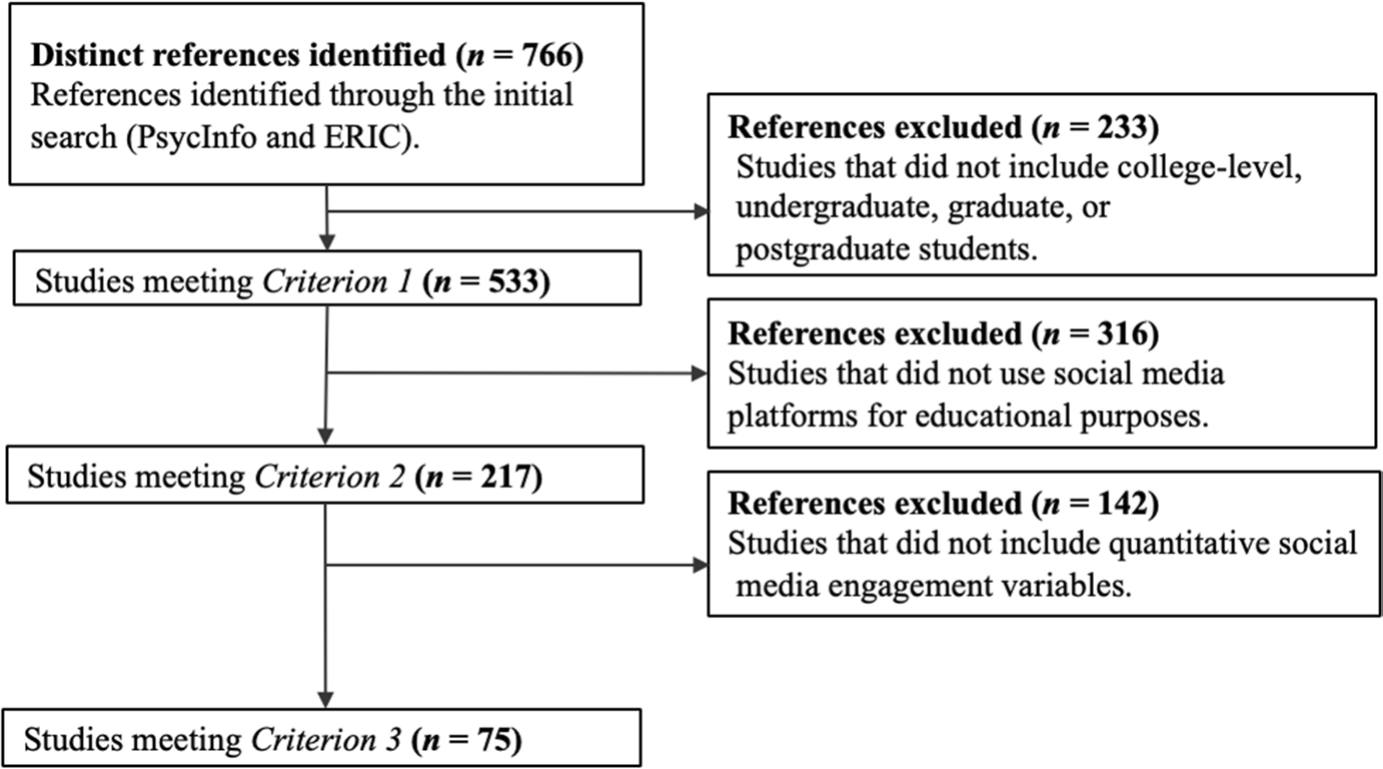
Study Selection Flowchart

We included studies meeting the following inclusion criteria: (a) the study included college-level, undergraduate, graduate, or postgraduate students (Criterion 1), (b) the study used a social media platform for educational purposes (Criterion 2), and (c) the study included at least one social media engagement variable (Criterion 3). We screened the abstracts of the studies identified through the initial search to assess Criteria 1 and 2. We retrieved and processed the full manuscripts of studies meeting Criteria 1 and 2 for the purposes of verification and for evaluating Criterion 3. The initial search returned 766 distinct references. We implemented inclusion criteria sequentially. Figure 1 presents a detailed record of the implementation of the inclusion criteria (see also Supplementary Online Material, Table A). Seventy-five studies met all inclusion criteria and proceeded to the data extraction phase.

Three raters participated in the study. The first rater applied inclusion criteria to all references originally retrieved (ATR). For the purposes of evaluating interrater agreement during the screening of references, two secondary raters (APG, JVO) independently applied inclusion criteria to the first 400 references (52% of all references). The primary and secondary raters applied the inclusion criteria to all 400 references identically, resulting in an interrater agreement of 100%. We also computed interrater agreement for the data extraction process for the selected studies. Specifically, a secondary rater (APB) extracted the 11 target variables of all selected studies (total number of participants, participants' age, participants' gender, field of study, social media platform utilized, country, course level, study design, social media engagement, quantitative outcome variables, qualitative outcome variables). Raters used closed lists (dropdown menus) to input each variable extracted onto the study database. We computed the interrater agreement of the data extraction process for each selected reference as the number of agreements plus disagreements divided by 11 and converted this ratio into a percentage. We then computed the mean interrater agreement across all selected references. An agreement was defined as both raters extracting the exact same piece of information for a target variable of a selected reference. A disagreement was defined as the two raters extracting different information for a target variable of a selected reference. Overall, there were four disagreements pertaining to four distinct selected references. Therefore, the mean interrater agreement of the data extraction process was 99.5% (range, 90.9–100%). The data extracted by the primary rater was used during the analyses.

The use of multiple databases, independent raters, and an interrater agreement process was intended to minimize the risk of bias during the selection of references. The current systematic review adheres to the PRISMA statement for reporting systematic reviews (see Supplementary Online Information). The database resulting from the systematic review has been made available via Figshare (Virues-Ortegaet al., 2022a).

### Data Extraction

The following variables were extracted from all studies meeting the inclusion criteria.

#### Number, Age, Gender, and Country of Participants

We recorded the total number of participants as well as their age, gender, and educational level (high school, college, master’s or PhD). We also recorded the country in which the study was conducted.

#### Field of Study

We recorded the field of study of all educational interventions. These were then classified according to an ad hoc category system. For example, research conducted with astronomy or physics students was categorized as "physical and life sciences", psychology and sociology students were categorized as "social sciences", second language students were included within "language and communication," and students in management of information and digital content were grouped under "computer and technology". Additional categories were used for "art," "business," and "professional courses."

#### Study Design

Studies simply using a questionnaire or interview at the end of the course were classified as "qualitative (retrospective)." Studies that implemented their assessments before and/or during the intervention were categorized as "qualitative (prospective)". Studies presenting correlation analyses for two or more variables at a given time point were classified as "observational." Studies using experimental designs were divided into two categories: "intervention (within subject),” for studies where all participants were exposed at least to a control and treatment or posttreatment condition; and "intervention (between groups)" when students were assigned to control and interventions groups. Randomization was not considered part of the classification process as none of the selected studies included random assignment.

#### Predictive and Outcome Variables

We also recorded predictive and outcome variables reported in the studies reviewed, including study time, academic achievement, user satisfaction, and personality constructs. Predictive and outcome variables were further classified as quantitative (objective and standardized tests) or qualitative (interviews, surveys, ad hoc questionnaires). Additional personal outcomes (e.g., motivation, sense of community, positive feelings) were included in a miscellaneous category.

#### Social Media Platforms and Engagement

We recorded the social media platform utilized in each study included in the review. Engagement could be evaluated with ad hoc interviews or surveys ("interview or survey”), standardized tests ("standardized test"), or quantitative engagement metrics ("behavioral”). The latter included discrete outcomes such as posting, commenting, and reaction frequencies, among others.

### Rational Process for Developing a Metrics Taxonomy

The development of the taxonomy of behavior-based metrics of social media engagement followed a three-step process of systematic review, classification, and rational extension. Completing the systematic review and obtaining a complete repertoire of the quantitative metrics of social media engagement in the existing literature was the initial step in the process of developing the taxonomy. Metrics were then classified by two key dimensions: (a) type (i.e., count-, time-, and topography-based), and (b) level of analysis (i.e., group, post, individual). The *type* dimension closely follows existing classifications of generalist behavioral observation metrics that often rely on the reoccurrence, time distribution, and topography of behavioral events (e.g., Johnston et al., 2019). For example, frequency is a count-based dimension, whereas latency is a time-based dimension under this classification. Metrics relying on more complex aspects of the event (e.g., length, meaning, use of emoticons) were considered topography-based metrics. The *level of analysis* is a result of the practical use of engagement metrics in social media, which may be reported at the level of the individual (e.g., commenting frequency of Student A), group (e.g., commenting frequency of Group A), or post (e.g., commenting frequency of Post A). Evidently, not all metrics could be practically implemented at all levels of analysis, for example, *posting frequency* could only be obtained at the group and individual levels, but not at the post level. A final step in this process involved extending the identified metrics by adding new exemplar metrics within the proposed categories. These additions have not been used in the literature yet but are conceivably practical in this context. For example, *reacting latency* is a time-based metric that is yet to be utilized in this literature.

The taxonomy was developed by consensus among the authors and is intended as an evidence-informed non-comprehensive repertoire of metrics. It should be noted that taxonomies proposed in psychology, and in behavior analysis in particular, often follow a rational process (e.g., Hackenberg 2018), and only rarely can a taxonomy be the result of a purely quantitative classification (e.g., Matthews et al., 1987).

## Results

### General Characteristics of Studies

Table 1 summarizes the results of the data extraction process. A pooled sample of 11,605 students participated in a total of 75 selected studies (age range, 17–60, 69.1% female). Social sciences (e.g., psychology and sociology) was the most common field of study among the studies reviewed (30.7%, *n* = 23), followed by language and communication (e.g., second language learning courses) (29.3%, *n* = 22), computer and technology (16%,* n* = 12), physical and life sciences (e.g., astronomy) (9.3%, *n* = 7), and vocational programs (e.g., digital content management) (5.3%, *n* = 4). The studies were geographically varied. There were missing values in some of the variables targeted for data extraction, including age of the students (*n* = 52), gender of the students (*n* = 42), course level (*n* = 1), and field of study (*n* = 3).

**Table 1:** Characteristics of Included Studies (*n* = 75)

Study	*n*	Age	Gender (% female)	Field of study	SMP	Country	Level	Study Design	Engagement metric	Outcome variables	
										Quantitative^a^	Qualitative
Abd-El-Aal and Steele (2017)	99	NR	88%	SS	F	Egypt	UG	Qualitative	SIT (ad hoc)	N/A	Satisfaction (ad hoc SIT)
Abney et al. (2018)	144	21	49.3%	B	T	US	UG	Observational	SIT (ad hoc)	N/A	Achievement (SIT)
Akcaoglu and Lee (2018)	62	adults	NR	PC	F	US	NU	Intervention (WS)	SIT (ad hoc)	N/A	Satisfaction (ad hoc SIT)
Al-Azawei (2019)	143	18–20	47.6	C&T	F	Iraq	UG	Observational	SIT (ad hoc)	N/A	Satisfaction (ad hoc SIT)
Albayrak & Yildirim(2015)	42	NR	NR	P&L	F	Turkey	UG	Qualitative	SIT (ad hoc)	N/A	Satisfaction (ad hoc SIT)
Alberth (2019)	64	NR	NR	L&C	F	Indonesia	UG	Intervention (WS)	SIT (ad hoc)	Achievement, writing skills (objective)	Other (SIT)
Alghazo & Nash (2017)	322	NR	34.2%	SS	W	Saudi Arabia	UG	Intervention (BG)	Behavioral (# missed sessions)	Achievement (objective)	N/A
Altunkaya and Topuzkanamış (2018)	96	NR	NR	SS	F	Turkey	UG	Intervention (BG)	SIT (ad hoc)	N/A	N/A
Arabacioglu and Akar-Vural (2014)	42	NR	61.9%	C&T	F	Turkey	UG	Intervention (BG)	SIT (ad hoc)	N/A	Other (SIT)
Bacile (2013)	86	NR	NR	B	F/T/I	US	UG	Intervention (WS)	Behavioral (# posts, likes)	N/A	N/A
Bahati (2015)	84	NR	28.6%	SS	F	Rwanda	G	Qualitative	SIT (ad hoc)	N/A	Satisfaction (ad hoc SIT)
Bajko et al. (2016)	76	18–40	64.5%	C&T	NR	Canada	UG	Intervention (BG)	SIT (ad hoc)	N/A	Satisfaction (ad hoc SIT)
Balcikanli (2015)	113	18–23	85%	L&C	F	Turkey	UG	Qualitative	SIT (ad hoc)	N/A	N/A
Carver (2019)	83	NR	NR	L&C	I	US	UG	Intervention (WS)	SIT (ad hoc)	N/A	Satisfaction (ad hoc SIT)
Chen (2015)	134	18–40	48.6	SS	F	Taiwan	UG	Intervention (WS)	SIT (ad hoc)	Achievement (objective)	Satisfaction (ad hoc SIT)
Clements (2015)	78	NR	NR	P&L	F	Canada	UG	Intervention (WS)	Behavioral (# posts, comments, likes)	Achievement (objective)	Other (SIT)
Daniels & Billingsley (2014)	20	19–34	NR	NR	F	US	UG	Intervention (WS)	Behavioral (# posts)	N/A	N/A
Delen, (2017)	58	NR	NR	SS	F	Turkey	UG	Intervention (WS)	Behavioral (# comments)	Achievement (objective)	N/A
Demirbilek (2015)	51	NR	80%	C&T	F	Turkey	UG	Qualitative	SIT (ad hoc)	N/A	Other (SIT)
Dizon & Thanyawatpokin (2018)	23	NR	NR	L&C	F	Japan	UG	Intervention (WS)	SIT (ad hoc)	N/A	N/A
Dougherty & Andercheck (2014)	170	NR	NR	SS	F	US	UG	Observational	Behavioral (# posts and likes)	Achievement, completed assignments (objective)	Satisfaction (ad hoc SIT)
Duncan & Barczyk (2016)	586	18- + 25	49.1%	PC	F	US	UG	Intervention (BG)	SIT (ad hoc)	N/A	Satisfaction (ad hoc SIT)
Ercoskun et al., 2019	1450	NR	68%	SS	NR	Turkey	UG	Observational	Behavioral (# of posts)	NA	NA
Evans (2014)	252	18–24	51%	PC	T	UK	UG	Qualitative	SIT (ad hoc)	N/A	N/A
Gamble & Wilkins (2014)	97	18	48.4%	L&C	F	Japan	UG	Qualitative	SIT (ad hoc)	N/A	Satisfaction (ad hoc SIT)
Giannikas (2019)	14	25–60	75%	L&C	F	Republic of Cyprus	UG	Qualitative	SIT (ad hoc)	N/A	Satisfaction (ad hoc SIT)
Goktalay (2015)	41	NR	86%	SS	F	Turkey	UG	Intervention (BG)	Standardized test (UTAUT)	N/A	Achievement (SIT)
Gordon (2016)	21	NR	NR	C&T	F	US	UG	Observational	Behavioral (# posts, comments, likes	N/A	N/A
Gregory et al. (2014)	78	NR	32.1%	PC	F	US	UG	Intervention (BG)	SIT (ad hoc)	N/A	Satisfaction and achievement (ad hoc SIT)
Gregory et al. (2016)	138	NR	NR	P&L	F	US	UG	Qualitative	SIT (ad hoc)	N/A	N/A
Guo et al. (2018)	129	18–24	NR	B	F	US	UG	Intervention (BG)	SIT (ad hoc)	N/A	Satisfaction (ad hoc SIT)
Harting (2017)	9	NR	NR	L&C	F	Japan	UG	Intervention (WS)	Behavioral (# posts)	Achievement (objective)	Satisfaction (ad hoc SIT)
Hennessy et al. (2016)	150	20 a 35	53.3%	P&L	F	UK	UG	Qualitative	SIT (ad hoc)	Achievement (objective)	Satisfaction (ad hoc SIT)
Hou et al. (2015)	50	NR	NR	C&T	F	Taiwan	UG	Observational	Behavioral (# posts and comments)	N/A	N/A
Ibarra (2018)	30	NR	NR	L&C	F	Ecuador	NU	Intervention (WS)	SIT (ad hoc)	Achievement (objective)	Satisfaction (ad hoc SIT)
Lee and Lee (2016)	108	NR	NR	NR	F	South Korea	NR	Intervention (BG)	Behavioral (semantic analysis metric for comments)	N/A	N/A
Luo (2018)	24	19–22	NR	SS	T	US	UG	Observational	Behavioral (# posts and characters per post)	N/A	N/A
Martínez-Cardama and Caridad-Sebastián (2019)	92	NR	NR	C&T	T	Spain	UG	Observational	Behavioral (# specific hashtags)	N/A	N/A
Miller (2013)	59	NR	NR	P&L	F	US	UG	Intervention (WS)	Behavioral (# posts and comments per student)	Achievement (objective)	N/A
Moghavvemi and Salarzadeh Janatabadi (2018)	170	NR	NR	P&L	F	Malaysia	UG	Intervention (WS)	Standardized test (UTAUT)	N/A	N/A
Montoneri (2015)	23	NR	NR	L&C	F	Taiwan	UG	Observational	Behavioral (# likes and views by post)	Achievement (objective)	Satisfaction (ad hoc SIT)
Montoneri (2017)	32	NR	NR	L&C	F	Taiwan	UG	Qualitative	SIT (ad hoc)	N/A	Other (SIT)
Naghdipour & Eldridge, (2016)	25	NR	52%	L&C	F	Turkey	UG	Observational	Behavioral (# comments and posts and comment-generating posts)	N/A	N/A
Narayan et al. (2019)	336	NR	NR	SS	T	New Zealand	UG	Intervention (WS)	Behavioral (# tweets, hashtags and posts)	N/A	Other (SIT)
Nazir and Brouwer (2019)	74	20–34	30%	NR	F	Netherlands	UG	Intervention (BG)	Behavioral (students and instructors # posts and comments)	N/A	Satisfaction (ad hoc SIT)
Nkhoma et al., (2015)	136	NR	NR	SS	F	Vietnam	UG	Intervention (WS)	SIT (ad hoc)	N/A	Achievement (ad hoc SIT)
Orawiwatnakul & Wichadee (2016)	82	NR	NR	L&C	F	Turkey	UG	Intervention (WS)	Behavioral (# posts)	Achievement (objective)	Satisfaction (ad hoc SIT)
Owens & Nussbaum (2017)	27	NR	93%	SS	T	US	UG	Intervention (WS)	Behavioral (# posts)	N/A	Other (SIT)
Pai et al. (2017a)	154	NR	NR	C&T	F	US	UG	Intervention (BG)	Behavioral (# posts, comments, likes)	N/A	N/A
Pai et al. (2017b)	142	18	NR	C&T	F	US	UG	Intervention (BG)	Behavioral (# posts, comments, likes; textual length of posts and comments)	N/A	N/A
Peeters and Pretorius (2020)	157	17–52	NR	L&C	F	Belgium	UG	Intervention (BG)	Behavioral (# posts across students)	N/A	N/A
Ping and Maniam (2015)	30	NR	NR	L&C	F	Malaysia	UG	Intervention (BG)	SIT (ad hoc)	Achievement (objective)	N/A
Popescu and Badea (2020)	74	22–40	23%	SS	T	Romania	UG	Intervention (BG)	Behavioral (# tweets)	N/A	N/A
Purnamasari (2019)	56	NR	NR	L&C	F	Indonesia	UG	Observational	SIT (ad hoc)	N/A	Satisfaction (ad hoc SIT)
Rahman et al. (2020)	108	16–35	54.2%	SS	F	US	UG	Observational	SIT (ad hoc	N/A	Satisfaction (ad hoc SIT)
Riady (2014)	1194	NR	NR	SS	F	Indonesia	UG	Qualitative	Behavioral (# posts, links, photos,events, updates, shared docs)	N/A	N/A
Rubrico & Hashim (2014)	59	19–27	98.3%	L&C	F	Malaysia	UG	Intervention (WS)	SIT (ad hoc)	N/A	Satisfaction (ad hoc SIT)
Saifudin et al. (2016)	58	22–26	57%	SS	F	Malaysia	UG	Intervention (WS)	SIT (ad hoc)	N/A	N/A
Schroeder and Greenbowe (2009)	128	NR	NR	C&T	F	US	UG	Observational	Behavioral (# posts)	N/A	N/A
Sheeran and Cummings (2018)	471	17–59	76.7%	SS	F	Australia	UG	Intervention (BG)	SIT (ad hoc)	N/A	Satisfaction (ad hoc SIT)
Shih (2011)	23	NR	78.3%	L&C	F	Taiwan	UG	Intervention (WS)	Behavioral (# comments and likes)	Achievement (objective)	Satisfaction (ad hoc SIT)
Sittiwong & Wongnam (2015)	38	NR	NR	C&T	F	Thailand	UG	Qualitative	SIT (ad hoc)	N/A	N/A
Slim & Hafedh, (2019)	102	NR	NR	L&C	F	Saudi Arabia	UG	Intervention (BG)	SIT (ad hoc)	Achievement (objective)	N/A
Teixeira & Hash (2017)	45	NR	NR	SS	T	US	UG	Qualitative	SIT (ad hoc)	N/A	Satisfaction (ad hoc SIT)
Tran (2016)	21	NR	14.3%	L&C	F	Vietnam	UG	Observational	Behavioral (# posts and likes)	Achievement (objective)	Satisfaction (ad hoc SIT)
Tucker (2015)	16	NR	75%	PC	F	US	UG	Intervention (BG)	SIT (ad hoc)	N/A	Other (SIT)
Tur & Marin (2015)	153	NR	NR	SS	T	Spain	UG	Observational	Behavioral (# tweets, retweets, and comments)	N/A	N/A
VanDoorn & Eklund (2013)	20	NR	NR	SS	F	Australia	UG	Qualitative	SIT (ad hoc)	N/A	Achievement (SIT)
Wang et al. (2013)	415	NR	63.2%	SS	F	Taiwan	UG	Observational	SIT (ad hoc)	Personality tests (Standarized)	N/A
Whittaker et al. (2014)	42	NR	NR	P&L	F	Australia	UG	Observational	Behavioral (# posts)	N/A	N/A
Wu et al., (2020)	24	NR	52.2%	C&T	F	Taiwán	UG	Observational	Behavioral (# posts and average of words)	N/A	N/A
Yagci (2015)	177	NR	54.2%	L&C	F	Iraq	UG	Qualitative	SIT (ad hoc)	N/A	N/A
Yu (2014)	NR	NR	NR	L&C	F	Taiwan	UG	Qualitative	Behavioral (# posts)	N/A	Satisfaction (ad hoc SIT)
Zhang and Lu (2014)	41	18–29	NR	L&C	F	Ireland	UG	Intervention (BG)	Behavioral (# characters)	Achievement (objective)	N/A

*B* Business, *BG* Between-groups design, *C&T* Computer and technology, *F* Facebook, *G* Graduate, *L&C* Language & communication, *N/A* Not applicable, *NR* Not reported, *NU* Non-university course, *P&L* Physical & life sciences, *PC* Professional courses, *SIT* Survey or interview, *SMP* Social media platform, SS Social sciences, *T* Twitter, *UG* College/Undergraduate, *UTAUT* User acceptance of information technology (Venkatesh et al., 2003), *W* WhatsApp, *WS* Within-subjects design

^a^If available, quantitative engagement metrics are reported in the column *Engagement metric*

Overall, 53.3% of studies (*n* = 40) evaluated an intervention mediated by a social media platform. Of these, 26.7% (*n* = 20) used a between-group design, and 26.7% (*n* = 20) used a pre-post within-subject design with no control group. None of the between-group studies was a randomized controlled trial. Observational or correlational studies that did not evaluate an intervention but conducted regression analyses of engagement and personal outcomes cross-sectionally were the second most frequent study design (24%, *n* = 18). The remaining studies were prospective qualitative studies that did not include a formal intervention or control group (21.3%, *n* = 16).

### Predictive and Outcome Variables

Some studies introduced social media platforms as an intervention intended to enhance academic achievement. Among intervention studies, 22.7% (*n* = 17) used pre-post academic achievement evaluated through ad hoc surveys, assignment marks, objective tests, and course grades. Sixteen of these intervention studies prospectively manipulated the introduction of a social media platform as a deliberate intervention. Finally, 11 studies (14.7%) evaluated both engagement and achievement with qualitative methods.

Some qualitative studies used ad hoc surveys to evaluate additional outcomes of the students’ educational experience while using the social media platform. Specifically, 28 studies evaluated student satisfaction using the social media platform (37.3%, *n* = 19), and five studies assessed the sense of community belonging (10.2%, *n* = 5). Additional outcomes (e.g., intrinsic motivation) were not observed more than twice in the pool of studies included in the review. The vast majority of included studies that used qualitative methods did not report any additional outcomes (see Table 1 for details).

### Social Media Platforms and Engagement

Approximately half of the studies selected (52%, *n* = 39) utilized ad hoc interviews and surveys to estimate the degree of social media participation among students, whereas thirty-three studies (44%) used some form of quantitative analysis of engagement based on the metrics provided by the social media platform (e.g., frequency of likes, comments, and posts). Table 2 summarizes the objective measures of social media engagement present in this literature. Specifically, Clements (2015), Gordon (2016), and Pai et al., (2017a, 2017b) computed the total number (frequency) of likes, comments, and posts from the group of students using the social network as a teaching and communication channel. In addition, eight studies used the frequency of posts and comments only (Hou et al., 2015; Lim, 2010; Luo, 2018; Miller, 2013; Naghdipour & Eldridge, 2016; Nazir & Brouwer, 2019; Peeters and Pretorius 2020; Wu et al., 2020). Miller (2013) departed from this trend by extracting the frequency of posts and comments per student as well as exploring posting immediacy. Luo (2018) and Wu et al., (2020) extracted the total number of characters composing each social media comment, while Nazir and Brouwer (2019) conducted a systematic theme analysis using the text of Facebook comments as samples. Bacile (2013), Dougherty and Andercheck (2014) and Tran (2016) collected the frequency of posts and likes, Shih (2011) the frequency of likes and comments, and Montoneri (2015) the frequency of views and likes per post. Finally, eight additional studies reported the frequency of posts as their single quantitative engagement outcome (Daniels & Billingsley 2014; Ercoskun et al., 2019; Harting, 2017; Orawiwatnakul & Wichadee 2016; Owens & Nussbaum 2017; Riady, 2014; Schroeder & Greenbowe, 2009; Whittaker et al., 2014; Yu, 2014).

**Table 2:** Objective Measures of Social Media Engagement in the Tertiary Education Literature

References	Platform	Level of analysis	Frequency			Other
			posts	Comments	Reactions	
Bacile (2013)	Facebook	Group	•		•	
Clements (2015)	Facebook	Group	•	•	•	
Daniels & Billingsley (2014)	Facebook	Group	•			
Dougherty & Andercheck (2014)	Facebook	Group	•		•	
Ercoskun et al., 2019	Facebook	Group	•			
Gordon (2016)	Facebook	Group	•	•	•	
Harting (2017)	Facebook	Group	•			
Hou et al. (2015)	Facebook	Group	•	•		
Lee and Lee (2016)	Facebook	Group	•			
Lim (2010)	Facebook	Group	•	•		
Luo (2018)	Facebook	Group	•	•		•
Martínez-Cardama and Caridad-Sebastián (2019)	Twitter	Group				•
Miller (2013)	Facebook	Student	•	•		
Montoneri (2015)	Facebook	Post			•	•
Naghdipour & Eldridge, (2016)	Facebook	Group	•	•		
Narayan et al. (2019)^a^	Twitter	Group	•	•	•	
Nazir and Brouwer (2019)	Facebook	Group	•	•		
Orawiwatnakul & Wichadee (2016)	Facebook	Group	•			
Owens & Nussbaum (2017)	Facebook	Group	•			
Pai et al. (2017a)	Facebook	Group	•	•	•	
Pai et al. (2017b)	Facebook	Group	•	•	•	
Peeters and Pretorius (2020)	Facebook	Group	•	•		
Popescu and Badea (2020)^a^	Twitter	Group	•			
Riady (2014)	Facebook	Group	•			•
Schroeder and Greenbowe (2009)	Facebook	Group	•			
Shih (2011)	Facebook	Group		•	•	
Tran (2016)	Facebook	Group	•		•	
Tur et al. (2015)^a^	Twitter	Group	•	•	•	
Whittaker et al. (2014)	Facebook	Group	•			
Wu et al., (2020)	Facebook	Group	•	•		•
Yu (2014)	Facebook	Group	•			
Zhang & Lu (2014)	Facebook	Group		•		•

^a^Posts, comments, and reactions are considered equivalent to tweets, replies, and retweets, respectively

All above-mentioned studies utilized Facebook as their integrated social media platform. Four additional studies used Twitter; Tur and Marin (2015), Narayan et al. (2019), and Popescu and Badea (2020) extracted the frequency of tweets, retweets, and replies to tweets, while Martínez-Cardama and Caridad-Sebastián (2019) computed the total number of hashtags. Finally, Alghazo and Nash (2017) used the texting social media app WhatsApp and monitored class attendance and missed assignments (social media engagement outcomes were not reported). In addition, Goktalay (2015), and Moghavvemi and Salarzadeh Janatabadi (2018) used standardized tests as an indirect assessment of social media engagement.

## Discussion

The current study reviewed the literature on social media as aids to education with the end goal of proposing a taxonomy of behavioral outcomes that could be utilized in experimental, translational, and applied research. Specifically, we reviewed the engagement metrics reported in studies using social media platforms as a learning channel in tertiary education. While most of the studies reviewed were qualitative, the systematic review provided a solid basis for a taxonomy including frequency- and time-based outcomes.

Our descriptive analysis showed that most studies utilized ad hoc surveys to document social media engagement and satisfaction (e.g., Gregory et al., 2014), while a minority of studies focused on objective engagement indicators including posts, comments, and reactions (e.g., Peeters & Pretorius 2020). Most interventional studies evaluated the addition of a social media platform on student satisfaction (Akcaoglu & Lee 2018), while few focused on objective or standardized academic achievement outcomes. For example, Dougherty & Andercheck (2014) correlated engagement, as estimated by the frequency of Facebook posts and reactions, with objective academic performance evaluated through weekly multiple-choice tests. Interventional studies followed pre-post within-subject and between-groups designs. Control groups were either withdrawn from the possibility of interacting through a target social media platform (Alghazo & Nash, 2017) or were exposed to a passive course instructor (Peeters & Pretorius 2020). Controlled studies lacked randomization (no RCTs were identified). Specifically, participants in experimental groups were often offered a choice to participate in a social media platform as part of their course, while those in the control group underwent the usual course format without a social media channel to enhance peer-to-peer or instructor-to-student interaction (e.g., Gregory et al., 2014).

Overall, this literature offers a limited picture of the quantitative engagement responses that could be recorded from social media platforms. Objectively defined engagement responses involved primarily posting and reacting frequency, whereas more sophisticated time-based or event-related outcomes were rarely explored (e.g., comment latency, percentage of individuals posting). In a notable exception, Miller (2013) studied posting frequency and posting immediacy. Posting immediacy may be defined as the time elapsed from a target instructor post to a post-related student response. Textual analysis methodology, including theme detection and sentiment analysis, which have become common social media research methods (Angus, 2017; Thelwall, 2017), were rare occurrences within this literature. Nazir and Brouwer (2019) illustrate an exception to this trend by qualitatively analyzing 67 post transcripts and comments made by both students and instructors in a Facebook group over the course of eight weeks. The aim of the textual analysis was to classify posts and comments according to three categories, “social presence,” “cognitive presence,” and “teaching presence.”

As per our taxonomy development process, we used the metrics documented in the literature (Table 2) as the basis to propose a non-compressive collection of behavior-based engagement metrics (Table 3). This summary is provided as a sample of relevant and intuitive metrics and is not intended as an exhaustive collection of all possible outcomes. Some of the proposed metrics are yet to be utilized in empirical studies. Existing metrics were divided into three categories, those based on the frequency or count of responses (count-based), those derived from the timing of the response (time-based), and those involving the automated analyses of the response length, content, and semantics (topography-based or text analysis). Some of these metrics can be applied to an individual, group, or post as level of analysis. For example, *commenting frequency* (i.e., total number of comments over a period of time), could refer to a group (i.e., total number of comments made by a group of individuals over a period of time), an individual (i.e., total number of comments made by an individual over a period of time), or a post (i.e., total number of comments by any individual responding to a particular post over a period of time).

**Table 3:** A proposed taxonomy of quantitative metrics of social media engagement

Metric & level of analysis	Definition & example reference if available	
Count-based	Posting frequency _G, I, P_	Total number of posts over a period of time (e.g., week, semester). Pai et. al. (2017a)
	Commenting frequency _G, I, P_	Total number of comments over a period of time (e.g., week, semester). Naghdipour & Eldridge, (2016)
	Reacting frequency _G, I, P_	Total number of reactions over a period of time (e.g., week, semester). Montoneri (2015)
	Percentage individuals posting _G_	Ratio of the total number of students publishing to the total number of posts
	Percentage individuals commenting _G, P_	Ratio of the total number of students commenting to the total number of comments in the study group
	Student-instructor posting ratio _P_	Ratio between the number of posts published by a specific group of people (e.g., students) and the total number of posts published (e.g., students and faculty members). Nazir and Brouwer (2019)
	Student-instructor commenting ratio _P_	Ratio between the number of comments posted by a specific group of people (e.g., students) and the total number of comments posted (e.g., students and faculty members). Nazir and Brouwer (2019)
	Percentage individuals reacting _P_	Ratio between the number of reactions posted by a specific group of people (e.g., students) and the total number of reactions posted (e.g., students and faculty)
Time-based	Commenting latency _G, I, P_	Time elapsed between the appearance of a post and the appearance of a comment
	Reacting latency _G, I, P_	Time elapsed between the appearance of a post and the appearance of a reaction to that post
	Posting inter-response time _G, I_	Time elapsed between successive posts. Lindström et al. (2021)
	Commenting inter-response time _I, P_	Time elapsed between the publication of a comment made by a person and the next comment
	Reacting inter-response time _I, P_	Time elapsed between the reaction published by one person to the next reaction
Text analysis	Post length _G, I, P_	Total number of characters in a post. Pai et al., (2017a, 2017b)
	Comment length _G, I, P_	Total number of characters in a comment. Pai et al., (2017a, 2017b)
	Theme analysis _G, I, P_	Occurrence and co-occurrence of words appearing in a text using an algorithmic process generated by software. Angus (2017)
	Sentiment analysis _G, I, P_	Total occurrence of terms analyzed by lexicon algorithms using software to detect sentiment-related patterns. Thelwall (2017)

*I* Individual, *G* Group, *P* Post

*Posting frequency* provides yet another example of a count-based metric, which may be defined as the total number of posts over a period of time (e.g., week, semester) and could be applicable to a group or an individual (Table 3). For example, Pai et. al. (2017a) monitored the instructor-led interactions in a Facebook group consisting of 150 biology students over a period of eight weeks. Authors extracted the total number of monthly posts by both students and instructors. The results of their descriptive analysis indicated that spontaneous student posting increased gradually over time (for an individual-level analysis of posting frequency see for example Tran, 2016). Posting frequency is significant in that, unlike commenting and reacting, reveals a spontaneous (unprompted) engagement with the discussion topic. Posting frequency can also be computed as a relative measure (i.e., total number of posts made by an individual or subgroup of individuals as a fraction of the total number of posts in the whole group or a different subgroup). Relative posting frequency may be used to obtain valuable information such as peer-to-peer or instructor-to-peer subgroup interaction or to provide data and compare the frequency of participation between subgroups within a wider social media group. These outcomes could also inform the correlation between engagement and academic performance or the effects on participation of an instructor-led intervention directed to a particular subgroup. An example of relative posting frequency can be found in Nazir and Brouwer (2019). The authors obtained the percentage of total posts and comments, including those specifically made by students and moderators in a Facebook group. However, relative measures are rarely used in the literature.

Time-based measures require additional attention. We highlight two common behavior dimensions yet to be explored as part of a quantitative analysis of engagement responses in education: latency and inter-response time (e.g., Rohrer & Wixted, 1994). Comment latency can be obtained by calculating time elapsed between a post or comment and its response. This parameter can be obtained for an individual (e.g., mean commenting latency of an individual), group (e.g., mean commenting latency of a group) or post (e.g., mean commenting latency of comments to a post). An example of the use of comment latency on social networks can be found in Lindström et al. (2021), who used this metric to evaluate the effects of receiving feedback in the form of “likes” from other participants. Comment latency allowed Lindström et al. to demonstrate that the posting behavior of participants has operant characteristics and is causally influenced by social rewards. Even though the study did not have an educational component, Lindström et al. pioneering quantitative analysis demonstrates the potential of engagement responses to reveal operant processes. On the other hand, inter-response time can be defined as the time that elapses between two comments (comment inter-response time) or two reactions (reaction inter-response time) made by an individual. Inter-response time describes the pace at which a behavior is performed, which may be informative in online environments where receiving positive responses may be in part a function of response omission (e.g., an instructor may be more likely to respond favorably to posts from students that have not participated recently in a social media group).

Most of the proposed metrics are widely used in applied behavior analysis, specifically when evaluating teaching procedures for students with and without developmental disability. For example, latency has been used to monitor the time elapsed from the presentation of an instruction to the initiation of a relevant response (Koegel et al., 2010). Frequency, defined as the total number of times a behavior occurs (Kubina & Lin, 2008), is also widely used in educational settings (Bishop et al., 2020). Frequency provides information on how often target responses are met being a critical outcome for self-paced and fluency-based teaching–learning paradigms (Kubina & Morrison, 2000). It is likely that these metrics that have already demonstrated their effectiveness on educational contexts will also prove useful in the rapidly expanding field of educational applications of social media. In the current review we identified only six studies that quantitatively measured engagement using frequency (i.e., total number of posts and reactions) concurrently evaluated academic performance. For example, Shih (2011) and Montoneri (2015) obtained measures before and after completing questionnaires based on a 5-point Likert scale assessing content, organization, structure, and orthographic content. By contrast, Alghazo and  Nash (2017), Dougherty and Andercheck (2014), Miller (2013), and Tran (2016) used final exams grades or course assignment marks to estimate academic performance. The evidence available still portrays a much fragmentary picture on the potential relation between social media engagement and academic performance (meta-analyses remain impractical).

Text analysis provides an additional means for processing social media engagement inputs in educational contexts. Text length (i.e., post length or comment length) is among the most elementary text analysis metrics available. For example, Pai et al., (2017a, 2017b) found that character count was larger for posts than comments. However, the authors did not assess the association between textual length and academic performance. Secondly, theme and sentiment analysis may also have heuristic value in this context. Theme analysis allows for the automated processing of natural language in order to generate a hierarchy of topics, identify patterns of interest, and interpret communicative processes (Angus, 2017). There are multiple text analysis algorithms available (see, for example, Xu et al., 2022) that can be useful for processing large volumes of text extracted from social media and obtain thematic trends that would be impossible to obtain otherwise. In addition, textual analysis metrics can provide an indication of the social validity of interventions. For example, in a recent study by Anderson et al. (2021), thematic and textual analyses were conducted to assess the social validity of Likert-type behavioral-analytic scales. The more complex textual analysis metrics (i.e., sentiment and theme analysis) are yet to be implemented in the scientific literature on educational interventions using social media as a potential learning channel.

Lastly, sentiment analysis metrics can help to understand the role of emotional and motivational factors in communication processes (e.g., Thelwall, 2017). Various algorithms can estimate the intensity and quantity of sentiments expressed in a large volume of texts to ascertain sentiment patterns over time. A study by Ortigosa et al. (2014) demonstrated the possibility of obtaining useful data from Facebook posts to obtain information about learning experiences arising from interaction and posts generated in social networks. Data extraction was evaluated, although not the usefulness of the information obtained from the interactions. Sentiment analysis can also provide instructors with critical ongoing information about student engagement and performance (see, for example, Zhou & Jun-min, 2020).

Various limitations to the current analysis and its supporting literature should be noted. Our review identified limitations in the analyses commonly used to assess engagement.. Specifically, studies that assessed engagement often relied on ad hoc Likert-type questionnaires. Only a few studies reported behaviorally derived direct engagement responses (e.g., frequency of posting). In addition, over one-third of the studies reviewed were conducted in the context of second language learning courses (e.g., English as a second language, ESL), thereby limiting the diversity of fields of study sampled in this review. The motivation and social dynamics of vocational (e.g., ESL) versus academic courses may be fundamentally different. Third, while most studies focused on social media engagement (using qualitative and quantitative approaches), only a handful of studies concurrently monitored academic achievement and other relevant outcomes. Fourth, none of the studies reviewed included a control group or control condition that equated student exposure to instructor inputs. This limitation is not trivial, as equating instructor-led inputs and exposure to course content may be a critical methodological standard to ensure the relevance of the control group in future RCTs. Fifth, the data extraction process including the secondary observations were conducted by the authors, which were privy to the goals of the study and may have been subjected to bias.

Finally, the proposed taxonomy of social media engagement metrics is not comprehensive as it is intended only to illustrate the range of potential outcomes that could be utilized in this context. Moreover, the metrics proposed ex novo (without precedent in the literature) have not been validated in empirical studies. Validation studies have the potential of expanding the proposed taxonomy further. For example, indices known to provide the same information may be combined as part of summary metrics (e.g., combined engagement indices may result from adding views, comments, and reactions).

## Conclusion

The analysis of discrete engagement responses opens the door to human operant and applied behavior-analytic studies within this field. In addition, pre- and post-test measures of students' academic performance should be incorporated to confirm whether the increase in engagement may be a good predictor of academic achievement. Randomized controlled trials are also missing from this literature, which are critical to determine the effectiveness of educational interventions delivered through the social media channel. These efforts could help to establish and empirical basis for the now pervasive trend of integrating social media in higher education (Aldahdouh et al., 2020). This line of research could potentially help higher education institutions and educators to adopt social media platforms in the development of educational plans and teaching strategies. Finally, wider use of quantitative behaviorally based metrics is needed in order to further evaluate an operant learning model of online behavior in educational settings.

## Supplementary Information

Below is the link to the electronic supplementary material.

Supplementary file1 (DOCX 34 kb)

Supplementary file2 (DOCX 102 kb)
