# Supplementary material for: Quantitative Indices of Student Social Media Engagement in Tertiary Education: A Systematic Review and a Taxonomy
Source: J Behav Educ. 2023 Apr 12:1–29. Online ahead of print. doi: 10.1007/s10864-023-09516-6 (PMC10090751; doi:10.1007/s10864-023-09516-6)
Supplement: Supplementary file 2 — Supplementary file2 (DOCX 102 kb) [file 10864_2023_9516_MOESM2_ESM.docx]

*SUPPLEMENTARY ONLINE MATERIAL*

**Table A**

*Implementation of Inclusion Criteria*

| **Reference** | **Archival Reference** | **Criterion 1** | **Criterion 2** | **Criterion 3** | **Status** |
| --- | --- | --- | --- | --- | --- |
| Abd-El-Aal et al. (2017) | File 1, no. 99 | Met | Met | Met | Included |
| Abdullah et al. (2019) | File 2, no. 83 | Met | Not met | Met | Not included |
| Abe et al. (2013) | File 8, no. 31 | Not met | Not met | Not met | Not included |
| Abney et al. (2019) | File 3, no. 49 | Met | Met | Met | Included |
| Abramo (2016) | File 7, no. 47 | Not met | Not met | Not met | Not included |
| Abu-Alruz (2014) | File 4, no. 38 | Met | Not met | Not met | Not included |
| Abulibdeh (2013) | File 6, no. 91 | Met | Met | Not met | Not included |
| AbuSa'aleek (2015) | File 2, no. 72 | Met | Not met | Met | Not included |
| Acun (2020) | File 1, no. 83 | Met | Not met | Met | Not included |
| Acun (2020) | File 1, no. 90 | Met | Not met | Met | Not included |
| Adhikary et al. (2018) | File 2, no. 3 | Not met | Not met | Not met | Not included |
| Agbo et al. (2020) | File 1, no. 67 | Met | Not met | Met | Not included |
| Agozzino et al. (2014) | File 8, no. 60 | Not met | Not met | Not met | Not included |
| Aharony (2014) | File 4, no. 42 | Met | Not met | Not met | Not included |
| Ahmed et al. (2017) | File 4, no. 6 | Met | Not met | Not met | Not included |
| Akcaoglu et al. (2018) | File 2, no. 94 | Met | Met | Met | Included |
| Akman et al. (2018) | File 6, no. 47 | Met | Not met | Not met | Not included |
| Aksoy (2018) | File 4, no. 5 | Met | Not met | Not met | Not included |
| Al-Azawei (2019) | File 1, no. 100 | Met | Met | Met | Included |
| Al-Bahrani et al. (2017) | File 7, no. 39 | Not met | Not met | Not met | Not included |
| Al-Dheleai et al. (2017) | File 1, no. 94 | Met | Not met | Met | Not included |
| Al-marayeah et al. (2017) | File 4, no. 82 | Met | Not met | Not met | Not included |
| Al-Qaysi et al. (2020) | File 2, no. 100 | Met | Not met | Met | Not included |
| Alam et al. (2019) | File 1, no. 23 | Met | Met | Not met | Not included |
| Albayrak et al. (2015) | File 4, no. 97 | Met | Met | Met | Included |
| Alberth (2019) | File 2, no. 91 | Met | Met | Met | Included |
| Aldahdouh et al. (2020) | File 3, no. 79 | Not met | Not met | Not met | Not included |
| Alden (2017) | File 7, no. 14 | Not met | Not met | Not met | Not included |
| AlGhamdi et al. (2018) | File 3, no. 83 | Met | Not met | Not met | Not included |
| Alghamdi et al. (2019) | File 4, no. 19 | Met | Met | Not met | Not included |
| Alghazo et al. (2018) | File 4, no. 77 | Met | Met | Met | Included |
| Alhadabi et al. (2020) | File 1, no. 15 | Met | Not met | Met | Not included |
| Alharbi et al. (2018) | File 3, no. 73 | Met | Not met | Not met | Not included |
| Alillaiti (2018) | File 3, no. 56 | Not met | Not met | Not met | Not included |
| Aljehani (2019) | File 2, no. 37 | Met | Not met | Met | Not included |
| Allen (2013) | File 6, no. 97 | Not met | Not met | Not met | Not included |
| Alm (2015) | File 2, no. 31 | Met | Not met | Met | Not included |
| Alon et al. (2014) | File 8, no. 79 | Met | Met | Not met | Not included |
| Alruwaili et al. (2020) | File 1, no. 57 | Met | Not met | Not met | Not included |
| Alsaleem (2018) | File 1, no. 78 | Met | Met | Met | Included |
| Alt (2017a) | File 6, no. 17 | Met | Not met | Not met | Not included |
| Alt (2017b) | File 6, no. 73 | Met | Not met | Not met | Not included |
| Altin et al. (2018) | File 2, no. 85 | Met | Not met | Met | Not included |
| Altunkaya et al. (2018) | File 1, no. 79 | Met | Met | Met | Included |
| Amador et al. (2017) | File 3, no. 8 | Met | Not met | Not met | Not included |
| Amadu et al. (2018) | File 3, no. 98 | Met | Not met | Not met | Not included |
| Amin et al. (2016) | File 1, no. 82 | Met | Not met | Met | Not included |
| Anderson (2019) | File 2, no. 15 | Not met | Not met | Not met | Not included |
| Anderson et al. (2014) | File 8, no. 54 | Met | Met | Not met | Not included |
| Apostel (2015) | File 7, no. 3 | Met | Not met | Not met | Not included |
| Arabacioglu (2014) | File 2, no. 92 | Met | Met | Met | Included |
| Aragon et al. (2012) | File 3, no. 65 | Met | Not met | Not met | Not included |
| Arendale (2017) | File 4, no. 96 | Not met | Met | Not met | Not included |
| Arias Chávez et al. (2018) | File 1, no. 14 | Not met | Not met | Not met | Not included |
| Armstrong et al. (2017) | File 6, no. 16 | Met | Not met | Not met | Not included |
| Arquero et al. (2017) | File 6, no. 99 | Met | Met | Not met | Not included |
| Arshad et al. (2018) | File 3, no. 69 | Met | Not met | Not met | Not included |
| Arslantas et al. (2019) | File 2, no. 70 | Met | Not met | Met | Not included |
| Arteaga et al. (2019) | File 1, no. 35 | Met | Not met | Met | Not included |
| Aslan et al. (2018) | File 3, no. 86 | Met | Not met | Not met | Not included |
| Atkins et al. (2017) | File 1, no. 9 | Not met | Not met | Met | Not included |
| Atwong (2015) | File 7, no. 1 | Not met | Met | Not met | Not included |
| Aubry (2013) | File 4, no. 40 | Met | Not met | Not met | Not included |
| Auxier (2020) | File 2, no. 22 | Not met | Not met | Met | Not included |
| Bacile (2013) | File 8, no. 89 | Met | Met | Met | Included |
| Bagarukayo (2018) | File 4, no. 66 | Met | Met | Not met | Not included |
| Bagarukayo et al. (2016) | File 2, no. 5 | Met | Met | Not met | Not included |
| Bagci et al. (2020) | File 1, no. 70 | Met | Not met | Not met | Not included |
| Bahati (2015) | File 2, no. 24 | Met | Met | Met | Included |
| Baim (2016) | File 7, no. 9 | Met | Met | Not met | Not included |
| Baim (2017) | File 4, no. 31 | Not met | Not met | Not met | Not included |
| Bajko et al. (2016) | File 4, no. 26 | Met | Met | Met | Included |
| Baker (2013) | File 5, no. 15 | Met | Not met | Not met | Not included |
| Baker et al. (2016) | File 3, no. 52 | Met | Not met | Not met | Not included |
| Bal et al. (2015) | File 7, no. 30 | Met | Met | Not met | Not included |
| Balakrishnan et al. (2017) | File 7, no. 65 | Met | Not met | Not met | Not included |
| Balcikanli (2015) | File 2, no. 56 | Met | Met | Met | Included |
| Baltaci (2019) | File 2, no. 81 | Met | Not met | Met | Not included |
| Balzhiser et al. (2011) | File 8, no. 86 | Met | Met | Not met | Not included |
| Bamberger et al. (2020) | File 3, no. 54 | Not met | Not met | Not met | Not included |
| Barczyk et al. (2012) | File 8, no. 71 | Met | Met | Not met | Not included |
| Barczyk et al. (2017) | File 1, no. 25 | Met | Met | Met | Included |
| Barden (2012) | File 5, no. 1 | Met | Met | Not met | Not included |
| Barnes et al. (2015) | File 4, no. 3 | Met | Not met | Not met | Not included |
| Barnett et al. (2011) | File 7, no. 75 | Met | Not met | Not met | Not included |
| Barquero et al. (2018) | File 2, no. 51 | Met | Met | No Met | Not Included |
| Barrot (2016) | File 3, no. 97 | Met | Met | Not met | Not included |
| Barrot (2018) | File 2, no. 29 | Not met | Not met | Not met | Not included |
| Bassili (2008) | File 9, no. 115 | Met | Not met | Not met | Not included |
| Battles et al. (2011) | File 8, no. 56 | Not met | Not met | Not met | Not included |
| Bedford (2019) | File 2, no. 97 | Not met | Met | Not met | Not included |
| Belanger et al. (2014) | File 8, no. 26 | Met | Not met | Not met | Not included |
| Belkofer et al. (2011) | File 8, no. 95 | Not met | Not met | Not met | Not included |
| Belluci et al. (2019) | File 5, no. 20 | Not met | Not met | Not met | Not included |
| Beltran-Cruz et al. (2013) | File 6, no. 82 | Met | Met | Not met | Not included |
| Benko et al. (2016) | File 7, no. 50 | Not met | Met | Not met | Not included |
| Bennett et al. (2014) | File 8, no. 3 | Met | Not met | Not met | Not included |
| Bennett et al. (2017) | File 7, no. 76 | Not met | Not met | Not met | Not included |
| Bennett et al. (2020a) | File 3, no. 32 | Met | Not met | Not met | Not included |
| Bennett et al. (2020b) | File 3, no. 77 | Not met | Not met | Not met | Not included |
| Berdanier et al. (2020) | File 3, no. 11 | Not met | Not met | Not met | Not included |
| Bessenoff (2006) | File 9, no. 103 | Met | Not met | Not met | Not included |
| Best et al. (2011) | File 3, no. 15 | Not met | Met | Not met | Not included |
| Best et al. (2018) | File 6, no. 5 | Met | Not met | Not met | Not included |
| Bharucha (2018) | File 6, no. 29 | Met | Not met | Not met | Not included |
| Bhatnagar et al. (2020) | File 1, no. 71 | Met | Not met | Met | Not included |
| Bigelow et al. (2016) | File 7, no. 63 | Met | Not met | Not met | Not included |
| Bilgin et al. (2018) | File 3, no. 26 | Met | Not met | Not met | Not included |
| Bindra et al. (2020) | File 1, no. 53 | Met | Not met | Met | Not included |
| Black et al. (2020) | File 3, no. 13 | Not met | Not met | Not met | Not included |
| Blankenship (2011) | File 8, no. 28 | Met | Not met | Not met | Not included |
| Bodnar et al. (2011) | File 1, no. 5 | Not met | Not met | Not met | Not included |
| Bolat (2018) | File 3, no. 6 | Met | Not met | Met | Not included |
| Boon et al. (2009) | File 6, no. 4 | Not met | Not met | Not met | Not included |
| Bor (2014) | File 8, no. 82 | Met | Met | Not met | Not included |
| Borch (2019) | File 2, no. 20 | Met | Met | Not met | Not included |
| Bowen et al. (2017) | File 4, no. 11 | Met | Not met | Not met | Not included |
| Bozanta et al. (2017) | File 4, no. 78 | Met | Not met | Not met | Not included |
| Brech et al. (2017) | File 1, no. 19 | Not met | Not met | Met | Not included |
| Bresmer et al. (2015) | File 8, no. 34 | Not met | Met | Not met | Not included |
| Brew et al. (2013) | File 3, no. 60 | Met | Not met | Not met | Not included |
| Briggs (2013) | File 7, no. 100 | Not met | Not met | Not met | Not included |
| Brocato et al. (2015) | File 7, no. 64 | Not met | Not met | Not met | Not included |
| Brookbank (2015) | File 7, no. 61 | Met | Not met | Not met | Not included |
| Brown et al. (2016) | File 7, no. 52 | Met | Not met | Not met | Not included |
| Brown et al. (2020) | File 3, no. 53 | Met | Met | Not met | Not included |
| Bruneel et al. (2013) | File 5, no. 36 | Met | Not met | Not met | Not included |
| Bryant et al. (2014) | File 6, no. 10 | Met | Not met | Not met | Not included |
| Bugeja (2006) | File 7, no. 33 | Not met | Not met | Not met | Not included |
| Bukhari et al. (2018) | File 2, no. 54 | Met | Not met | Met | Not included |
| Bulut et al. (2019) | File 2, no. 88 | Met | Not met | Not met | Not included |
| Burkhart et al. (2017) | File 3, no. 100 | Met | Not met | Not met | Not included |
| Buus (2012) | File 7, no. 58 | Met | Met | Not met | Not included |
| Buzzetto-More et al. (2015) | File 7, no. 13 | Met | Not met | Not met | Not included |
| Cabrera et al. (2017) | File 6, no. 7 | Not met | Not met | Not met | Not included |
| Callaghan et al. (2016) | File 3, no. 88 | Not met | Not met | Met | Not included |
| Cameron (2010) | File 9, no. 110 | Not met | Not met | Not met | Not included |
| Campbell et al. (2019) | File 4, no. 22 | Met | Met | Not met | Not included |
| Cao et al 2019 | File 3, no. 87 | Met | Not met | Not met | Not included |
| Cao et al. (2013) | File 8, no. 97 | Not met | Met | Not met | Not included |
| Carmichael et al. (2014) | File 6, no. 2 | Not met | Not met | Not met | Not included |
| Carnevale (2006) | File 9, no. 106 | Not met | Not met | Not met | Not included |
| Carreon et al. (2016) | File 5, no. 6 | Not met | Not met | Not met | Not included |
| Cartner et al. (2017) | File 4, no. 15 | Not met | Met | Not met | Not included |
| Carver (2019) | File 2, no. 41 | Met | Met | Met | Included |
| Celebi (2018) | File 1, no. 85 | Met | Met | Not met | Not included |
| Celik (2020) | File 2, no. 64 | Met | Not met | Met | Not included |
| Celik et al. (2015) | File 4, no. 4 | Met | Not met | Not met | Not included |
| Cevik et al. (2014) | File 4, no. 73 | Met | Met | Not met | Not included |
| Chai et al. (2016) | File 7, no. 49 | Met | Met | Not met | Not included |
| Chaka et al. (2020) | File 1, no. 41 | Met | Met | Met | Included |
| Chalupa (2015) | File 7, no. 4 | Not met | Not met | Not met | Not included |
| Chan (2017) | File 7, no. 56 | Met | Not met | Not met | Not included |
| Charlton et al. (2009) | File 6, no. 89 | Met | Not met | Not met | Not included |
| Charteris et al. (2018) | File 2, no. 68 | Met | Met | Not met | Not included |
| Chatten et al. (2016) | File 7, no. 20 | Not met | Not met | Not met | Not included |
| Chen (2015) | File 2, no. 50 | Met | Met | Met | Included |
| Chen et al. (2012) | File 7, no. 42 | Not met | Not met | Not met | Not included |
| Chen et al. (2014) | File 8, no. 42 | Not met | Not met | Not met | Not included |
| Cheng et al. (2016) | File 3, no. 25 | Met | Met | Not met | Not included |
| Childers (2017) | File 3, no. 96 | Met | Met | Not met | Not included |
| Childers et al. (2016) | File 7, no. 8 | Not met | Not met | Not met | Not included |
| Chromey et al. (2016) | File 7, no. 27 | Met | Not met | Not met | Not included |
| Chugh et al. (2018) | File 1, no. 63 | Not met | Not met | Not met | Not included |
| Churcher et al. (2014) | File 6, no. 38 | Met | Met | Not met | Not included |
| Clark et al. (2017) | File 7, no. 57 | Met | Not met | Not met | Not included |
| Clements (2015) | File 2, no. 96 | Met | Met | Met | Included |
| Cochrane et al. (2013a) | File 8, no. 29 | Met | Met | Not met | Not included |
| Cochrane et al. (2013b) | File 8, no. 38 | Met | Met | Not met | Not included |
| Cochrane et al. (2013c) | File 8, no. 50 | Met | Met | Not met | Not included |
| Coetzee et al. (2019) | File 4, no. 9 | Met | Not met | Not met | Not included |
| Connell (2009) | File 6, no. 6 | Met | Not met | Not met | Not included |
| Connolly (2014) | File 8, no. 11 | Not met | Not met | Not met | Not included |
| Connolly et al. (2019) | File 2, no. 21 | Met | Not met | Not met | Not included |
| Constantinides (2011) | File 9, no. 117 | Not met | Not met | Not met | Not included |
| Cooke (2017) | File 7, no. 78 | Met | Not met | Not met | Not included |
| Cowley (2017) | File 7, no. 84 | Not met | Met | Not met | Not included |
| Crews et al. (2012) | File 5, no. 42 | Not met | Not met | Not met | Not included |
| Cru (2015) | File 4, no. 51 | Met | Not met | Not met | Not included |
| Cuesta et al. (2016) | File 3, no. 94 | Met | Not met | Not met | Not included |
| Cumberledge (2020) | File 2, no. 80 | Not met | Not met | Met | Not included |
| Curro et al. (2018) | File 3, no. 70 | Not met | Not met | Not met | Not included |
| Curtis et al. (2019) | File 1, no. 39 | Met | Not met | Not met | Not included |
| da Silva et al. (2014) | File 4, no. 84 | Met | Met | Not met | Not included |
| Dabbagh et al. (2012) | File 8, no. 48 | Not met | Not met | Not met | Not included |
| Dabner (2012) | File 1, no. 7 | Not met | Not met | Met | Not included |
| Dahdal (2020) | File 2, no. 98 | Met | Not met | Met | Not included |
| Daniels et al. (2014) | File 1, no. 34 | Met | Met | Met | Included |
| Datko (2019) | File 1, no. 81 | Met | Not met | Not met | Not included |
| Daugird et al. (2015) | File 7, no. 26 | Not met | Not met | Not met | Not included |
| Davidovitch et al. (2018) | File 1, no. 31 | Met | Met | Met | Included |
| Davis et al. (2015) | File 7, no. 40 | Met | Met | Not met | Not included |
| Davis et al. (2015)b | File 8, no. 68 | Not met | Not met | Not met | Not included |
| DeAndrea et al. (2012) | File 8, no. 58 | Met | Not met | Not met | Not included |
| Deans (2012) | File 8, no. 33 | Met | Met | Not met | Not included |
| del Rocio Bonilla et al. (2020) | File 3, no. 48 | Not met | Not met | Met | Not included |
| Delello et al. (2015) | File 8, no. 98 | Met | Met | Not met | Not included |
| Demir (2018) | File 6, no. 90 | Met | Met | Not met | Not included |
| Demirbilek (2015) | File 1, no. 59 | Met | Met | Met | Included |
| Delen (2017) | File 1, no. 75 | Met | Met | Met | Included |
| Dennen et al. (2017) | File 3, no. 45 | Met | Not met | Not met | Not included |
| Dennen et al. (2020) | File 1, no. 69 | Not met | Not met | Not met | Not included |
| DePew (2011) | File 1, no. 61 | Met | Not met | Not met | Not included |
| Dincer et al. (2019) | File 2, no. 6 | Met | Not met | Met | Not included |
| Dizon (2010) | File 9, no. 102 | Not met | Met | Not met | Not included |
| Dizon (2016) | File 3, no. 2 | Met | Met | Not met | Not included |
| Dizon et al. (2016) | File 2, no. 99 | Met | Met | Met | Included |
| Doak (2011) | File 8, no. 51 | Not met | Not met | Not met | Not included |
| Dogan et al. (2018) | File 1, no. 93 | Met | Met | Not met | Not included |
| Dogari et al. (2019) | File 2, no. 82 | Met | Not met | Not met | Not included |
| Donelan (2016) | File 7, no. 68 | Not met | Not met | Not met | Not included |
| Donlan (2014) | File 4, no. 35 | Met | Not met | Not met | Not included |
| Dougherty et al. (2014) | File 4, no. 91 | Met | Met | Met | Included |
| Downes (2007) | File 6, no. 55 | Not met | Not met | Not met | Not included |
| Dragseth (2020) | File 3, no. 17 | Not met | Not met | Not met | Not included |
| Duncan et al. (2016) | File 2, no. 34 | Met | Met | Met | Included |
| Dyson et al. (2015) | File 4, no. 29 | Met | Met | Not met | Not included |
| Eaton et al. (2012) | File 7, no. 5 | Not met | Not met | Not met | Not included |
| Eberhardt (2007) | File 6, no. 35 | Not met | Not met | Not met | Not included |
| Edmiston (2016) | File 7, no. 21 | Not met | Not met | Not met | Not included |
| Ekoc (2014) | File 2, no. 32 | Met | Met | Not met | Not included |
| Elavsky (2013) | File 8, no. 5 | Not met | Not met | Not met | Not included |
| Ellefsen (2016) | File 2, no. 8 | Met | Not met | Not met | Not included |
| Enskat et al. (2017) | File 3, no. 4 | Met | Met | Not met | Not included |
| Eraslan et al. (2019) | File 2, no. 65 | Met | Not met | Not met | Not included |
| Ercoskun et al. (2019) | File 2, no. 45 | Met | Met | Met | Included |
| Erdem et al. (2014) | File 2, no. 71 | Met | Met | Met | Included |
| Erdogdu (2016) | File 2, no. 30 | Met | Not met | Met | Not included |
| Eroglu (2016) | File 2, no. 44 | Met | Not met | Met | Not included |
| Ersoz et al. (2018) | File 1, no. 24 | Not met | Met | Not met | Not included |
| Escobar-Rodriguez et al. (2014) | File 4, no. 43 | Met | Not met | Not met | Not included |
| Esgi (2016) | File 5, no. 19 | Met | Not met | Not met | Not included |
| Estrella ibarra (2018) | File 1, no. 45 | Met | Met | Met | Included |
| Evans (2014) | File 8, no. 93 | Met | Met | Met | Included |
| Fagioli et al. (2015) | File 6, no. 15 | Met | Not met | Not met | Not included |
| Fajardo Vizquerra et al. (2019) | File 1, no. 22 | Not met | Met | Met | Not included |
| Faulds et al. (2014) | File 8, no. 15 | Met | Met | Not met | Not included |
| Fenwick (2016) | File 7, no. 77 | Not met | Not met | Not met | Not included |
| Feria-Galicia (2011) | File 8, no. 41 | Not met | Not met | Not met | Not included |
| Fidan (2019) | File 1, no. 16 | Met | Not met | Met | Not included |
| Field et al. (2012) | File 7, no. 90 | Not met | Not met | Not met | Not included |
| Fife (2017) | File 4, no. 16 | Not met | Met | Not met | Not included |
| Filimowicz et al. (2017) | File 6, no. 22 | Met | Not met | Not met | Not included |
| Firat (2017) | File 1, no. 54 | Met | Not met | Met | Not included |
| Firat et al. (2017) | File 4, no. 24 | Not met | Not met | Not met | Not included |
| Fischbach et al. (2018) | File 6, no. 12 | Not met | Not met | Not met | Not included |
| Flores et al. (2020) | File 3, no. 67 | Met | Not met | Not met | Not included |
| Foos (2020) | File 1, no. 76 | Met | Not met | Not met | Not included |
| Fornara et al. (2019) | File 4, no. 98 | Met | Met | Not met | Not included |
| Friedman et al. (2013) | File 6, no. 93 | Not met | Not met | Not met | Not included |
| Friesen et al. (2012) | File 8, no. 90 | Not met | Not met | Not met | Not included |
| Frimming et al. (2011) | File 7, no. 31 | Met | Met | Not met | Not included |
| Frisby et al. (2016) | File 3, no. 57 | Met | Met | Not met | Not included |
| Fujita et al. (2017) | File 6, no. 42 | Met | Not met | Not met | Not included |
| Galan et al. (2015) | File 8, no. 70 | Met | Not met | Not met | Not included |
| Gamble et al. (2014) | File 2, no. 66 | Met | Met | Met | Included |
| Gammon et al. (2015) | File 7, no. 91 | Not met | Not met | Not met | Not included |
| Ganster et al. (2009) | File 6, no. 28 | Not met | Not met | Not met | Not included |
| Gavrin et al. (2017) | File 7, no. 28 | Met | Met | Not met | Not included |
| George (2011) | File 1, no. 37 | Not met | Not met | Not met | Not included |
| George et al. (2018) | File 5, no. 14 | Not met | Not met | Not met | Not included |
| Gerlich et al. (2010) | File 7, no. 87 | Met | Not met | Not met | Not included |
| Gettman et al. (2015) | File 2, no. 46 | Met | Not met | Met | Not included |
| Gharis et al. (2017) | File 6, no. 3 | Not met | Not met | Not met | Not included |
| Giannikas (2020) | File 1, no. 27 | Met | Met | Met | Included |
| Gilfoil et al. (2015) | File 5, no. 18 | Not met | Not met | Not met | Not included |
| Gilster et al. (2020) | File 3, no. 78 | Met | Met | Not met | Not included |
| Gin et al. (2016) | File 7, no. 18 | Met | Not met | Not met | Not included |
| Glenn (2015) | File 8, no. 6 | Not met | Not met | Not met | Not included |
| Glider et al., 2001 | File 9, no. 104 | Met | Not met | Not met | Not included |
| Goh et al. (2015) | File 8, no. 80 | Met | Not met | Not met | Not included |
| Goktalay (2015) | File 2, no. 78 | Met | Met | Met | Included |
| Gopalakrishnan et al. (2019) | File 3, no. 66 | Met | Not met | Not met | Not included |
| Gordon (2016) | File 3, no. 44 | Met | Met | Met | Included |
| Gorham et al (2012) | File 9, no. 119 | Not met | Not met | Not met | Not included |
| Green (2018) | File 3, no. 74 | Met | Not met | Not met | Not included |
| Greenwood (2012) | File 7, no. 32 | Not met | Not met | Not met | Not included |
| Grefory et al. (2016) | File 3, no. 39 | Met | Met | Met | Included |
| Gregory et al. (2014) | File 4, no. 79 | Met | Met | Met | Included |
| Griffin et al. (2020) | File 1, no. 38 | Met | Not met | Met | Not included |
| Gronbeck, 2000 | File 9, no. 108 | Not met | Not met | Not met | Not included |
| Guerin et al. (2020) | File 3, no. 22 | Not met | Not met | Not met | Not included |
| Guillaume et al. (2019) | File 4, no. 67 | Not met | Not met | Not met | Not included |
| Gulbahar et al. (2017) | File 4, no. 27 | Met | Met | Not met | Not included |
| Gundlach et al. (2015) | File 7, no. 79 | Met | Met | Not met | Not included |
| Guo et al. (2018) | File 6, no. 94 | Met | Met | Met | Included |
| Gupta et al. (2013) | File 6, no. 60 | Met | Not met | Not met | Not included |
| Halligan (2010) | File 9, no. 122 | Not met | Not met | Not met | Not included |
| Halliwell (2020) | File 3, no. 12 | Not met | Not met | Not met | Not included |
| Hamid et al. (2017) | File 7, no. 67 | Not met | Not met | Not met | Not included |
| Hamutoglu et al. (2020) | File 1, no. 43 | Met | Not met | Met | Not included |
| Hanson et al. (2011) | File 6, no. 14 | Met | Not met | Not met | Not included |
| Harting (2017) | File 1, no. 96 | Met | Met | Met | Included |
| Hashim et al. (2013) | File 3, no. 38 | Met | Met | Not met | Not included |
| Hassell et al. (2016) | File 4, no. 2 | Met | Not met | Not met | Not included |
| He et al. (2017) | File 6, no. 66 | Met | Not met | Not met | Not included |
| Heilig et al. (2019) | File 2, no. 53 | Not met | Not met | Not met | Not included |
| Hennessy et al. (2016) | File 7, no. 62 | Met | Met | Met | Included |
| Hermes (2008) | File 9, no. 107 | Not met | Not met | Not met | Not included |
| Hetz et al. (2015) | File 8, no. 66 | Met | Not met | Not met | Not included |
| Hickerson et al. (2017) | File 6, no. 44 | Met | Not met | Not met | Not included |
| Hoffman et al. (2014) | File 8, no. 19 | Met | Not met | Not met | Not included |
| Hong et al. (2015) | File 1, no. 87 | Met | Not met | Met | Not included |
| Hope (2016) | File 3, no. 23 | Met | Not met | Not met | Not included |
| Hottell et al. (2014) | File 8, no. 75 | Met | Not met | Not met | Not included |
| Hou et al. (2015) | File 4, no. 32 | Met | Met | Met | Included |
| Howard et al. (2017) | File 7, no. 85 | Met | Not met | Not met | Not included |
| Hughes et al. (2020) | File 3, no. 20 | Met | Not met | Not met | Not included |
| Hurt et al. (2012) | File 3, no. 81 | Met | Met | Not met | Not included |
| Hussain et al. (2018) | File 3, no. 72 | Met | Not met | Not met | Not included |
| Hutchens et al. (2014) | File 4, no. 46 | Met | Not met | Not met | Not included |
| Huwe (2011) | File 9, no. 113 | Not met | Not met | Not met | Not included |
| Huwe (2012) | File 6, no. 86 | Not met | Not met | Not met | Not included |
| Iqbal et al. (2016) | File 1, no. 1 | Met | Not met | Not met | Not included |
| Iredale et al. (2020) | File 3, no. 7 | Not met | Not met | Not met | Not included |
| Isbulan et al. (2020) | File 1, no. 4 | Met | Met | Met | Included |
| Islam (2018) | File 1, no. 21 | Met | Not met | Not met | Not included |
| Ismail et al. (2017) | File 4, no. 7 | Met | Not met | Not met | Not included |
| Ivala et al. (2012) | File 1, no. 62 | Met | Met | Not met | Not included |
| Iwamoto et al. (2020) | File 1, no. 80 | Met | Not met | Met | Not included |
| Izmirli (2017) | File 1, no. 8 | Met | Met | Not met | Not included |
| Jaffar (2014) | File 4, no. 33 | Met | Met | Not met | Not included |
| Jang (2015) | File 8, no. 13 | Met | Not met | Not met | Not included |
| Jebba et al. (2019) | File 1, no. 84 | Met | Not met | Met | Not included |
| Jin (2015) | File 4, no. 92 | Met | Met | Not met | Not included |
| Joan (2015) | File 2, no. 13 | Met | Not met | Met | Not included |
| Jocson (2015) | File 8, no. 45 | Met | Not met | Not met | Not included |
| Johannesen et al. (2019) | File 1, no. 40 | Met | Met | Not met | Not included |
| Johnson et al. (2017) | File 6, no. 100 | Met | Not met | Not met | Not included |
| Jones (2011) | File 7, no. 99 | Not met | Not met | Not met | Not included |
| Jones et al. (2019) | File 4, no. 14 | Met | Not met | Not met | Not included |
| Joseffson et al. (2016) | File 7, no. 88 | Met | Not met | Not met | Not included |
| Julien et al. (2018) | File 5, no. 39 | Not met | Not met | Not met | Not included |
| Jumaat et al. (2016) | File 3, no. 3 | Met | Met | Not met | Not included |
| Junco et al. (2010) | File 9, no. 105 | Not met | Not met | Not met | Not included |
| Kabilan (2016) | File 3, no. 95 | Met | Met | Not met | Not included |
| Kabilan et al. (2010) | File 6, no. 32 | Met | Not met | Not met | Not included |
| Kahveci (2015) | File 7, no. 54 | Met | Not met | Not met | Not included |
| Kalelioglu (2017) | File 1, no. 92 | Met | Met | Not met | Not included |
| Kaler et al. (2020) | File 3, no. 50 | Met | Not met | Not met | Not included |
| Karaa et al. (2016) | File 4, no. 23 | Met | Not met | Not met | Not included |
| Karademir et al. (2020) | File 1, no. 73 | Met | Not met | Met | Not included |
| Karaman (2019) | File 3, no. 30 | Met | Not met | Not met | Not included |
| Karanjakwut (2018) | File 1, no. 3 | Met | Met | Not met | Not included |
| Karl et al. (2011) | File 6, no. 37 | Met | Not met | Not met | Not included |
| Karpman et al. (2016) | File 7, no. 72 | Not met | Not met | Not met | Not included |
| Karsak (2016) | File 2, no. 86 | Met | Met | Not met | Not included |
| Kasuma (2017) | File 1, no. 36 | Met | Met | Not met | Not included |
| Kasuma et al. (2018) | File 6, no. 46 | Met | Not met | Not met | Not included |
| Katy (2020) | File 3, no. 46 | Met | Not met | Not met | Not included |
| Kelly (2018) | File 2, no. 67 | Met | Met | Not met | Not included |
| Kelm (2011) | File 8, no. 69 | Met | Met | Not met | Not included |
| Kettunen et al. (2013) | File 8, no. 9 | Not met | Not met | Not met | Not included |
| Khan et al. (2016) | File 7, no. 94 | Met | Not met | Not met | Not included |
| Khosrovani et al. (2016) | File 7, no. 41 | Met | Not met | Not met | Not included |
| Kidd et al. (2014) | File 8, no. 55 | Not met | Not met | Not met | Not included |
| Kilis et al. (2016) | File 5, no. 27 | Not met | Not met | Not met | Not included |
| Kim (2019) | File 4, no. 25 | Met | Met | Not met | Not included |
| Kim et al. (2014) | File 8, no. 94 | Met | Not met | Not met | Not included |
| Kim et al. (2016) | File 1, no. 13 | Met | Not met | Met | Not included |
| Kinchin et al. (2015) | File 8, no. 99 | Not met | Met | Not met | Not included |
| Kinsky (2015) | File 7, no. 92 | Met | Met | Not met | Not included |
| Kitchakarn (2016) | File 5, no. 41 | Met | Met | Not met | Not included |
| Ko (2019) | File 4, no. 71 | Met | Met | Not met | Not included |
| Kolek et al. (2008) | File 6, no. 51 | Met | Not met | Not met | Not included |
| Koles et al. (2012) | File 5, no. 32 | Met | Not met | Not met | Not included |
| Kooy (2016) | File 7, no. 10 | Not met | Not met | Not met | Not included |
| Koseoglu et al. (2016) | File 2, no. 74 | Met | Met | Not met | Not included |
| Koshkin et al. (2017) | File 6, no. 49 | Met | Not met | Not met | Not included |
| Kothari et al. (2016) | File 7, no. 74 | Met | Not met | Not met | Not included |
| Kramer et al. (2015) | File 4, no. 57 | Met | Not met | Not met | Not included |
| Krutka et al. (2017) | File 6, no. 85 | Met | Met | Not met | Not included |
| Kucuk et al. (2013) | File 3, no. 41 | Met | Met | Not met | Not included |
| Kumar et al. (2019) | File 4, no. 63 | Not met | Not met | Not met | Not included |
| Kurtz (2014) | File 4, no. 48 | Met | Met | Not met | Not included |
| Kwon et al. (2013) | File 5, no. 33 | Met | Not met | Not met | Not included |
| Lam (2012) | File 3, no. 10 | Met | Met | Not met | Not included |
| Lampe et al. (2011) | File 6, no. 71 | Met | Not met | Not met | Not included |
| Lansigan et al. (2016) | File 7, no. 60 | Met | Not met | Not met | Not included |
| LaRiviere et al. (2012) | File 8, no. 52 | Met | Not met | Not met | Not included |
| LaRoche et al. (2009) | File 6, no. 33 | Met | Not met | Not met | Not included |
| Larson (2018) | File 6, no. 75 | Not met | Not met | Not met | Not included |
| Lauricella (2019) | File 4, no. 20 | Not met | Not met | Not met | Not included |
| Lawson et al. (2011) | File 6, no. 88 | Met | Not met | Not met | Not included |
| Le et al. (2019) | File 5, no. 43 | Not met | Not met | Not met | Not included |
| Leafman et al. (2013) | File 6, no. 70 | Met | Not met | Not met | Not included |
| Lee (2018) | File 1, no. 18 | Met | Not met | Met | Not included |
| Lee et al. (2016) | File 2, no. 16 | Met | Met | Met | Included |
| Lemon et al. (2019) | File 1, no. 68 | Met | Met | Not met | Not included |
| Leppisaari et al. (2014) | File 8, no. 16 | Not met | Met | Not met | Not included |
| Levesque (2016) | File 7, no. 70 | Not met | Not met | Not met | Not included |
| Leyrer-Jackson et al. (2018) | File 6, no. 77 | Met | Not met | Not met | Not included |
| Li (2012) | File 8, no. 100 | Met | Met | Not met | Not included |
| Li et al. (2015) | File 8, no. 57 | Met | Not met | Not met | Not included |
| Lie (2013) | File 8, no. 62 | Met | Met | Not met | Not included |
| Liggett (2012) | File 8, no. 23 | Not met | Not met | Not met | Not included |
| Lilburn (2012) | File 8, no. 10 | Not met | Not met | Not met | Not included |
| Lim (2010) | File 4, no. 85 | Met | Met | Met | Included |
| Lin (2016) | File 3, no. 27 | Met | Met | Not met | Not included |
| Linder et al. (2016) | File 7, no. 35 | Met | Not met | Not met | Not included |
| Linh et al. (2016) | File 2, no. 73 | Not met | Met | Met | Not included |
| Lint (2013) | File 8, no. 4 | Met | Not met | Not met | Not included |
| Lint et al. (2013) | File 8, no. 35 | Met | Met | Met | Included |
| Linvill (2019) | File 4, no. 10 | Not met | Not met | Not met | Not included |
| Locatelli et al. (2012) | File 5, no. 34 | Met | Not met | Not met | Not included |
| Lohnes et al. (2016) | File 3, no. 31 | Met | Not met | Not met | Not included |
| Lou (2017) | File 6, no. 9 | Met | Met | Not met | Not included |
| Lou et al. (2012) | File 5, no. 7 | Met | Not met | Not met | Not included |
| Lovell et al. (2013) | File 5, no. 23 | Not met | Met | Not met | Not included |
| Lu et al. (2014) | File 6, no. 21 | Met | Not met | Not met | Not included |
| Lund (2019) | File 1, no. 89 | Not met | Not met | Met | Not included |
| Luo (2018) | File 6, no. 24 | Met | Met | Met | Included |
| Luo et al. (2020) | File 1, no. 46 | Not met | Not met | Not met | Not included |
| Madden et al. (2016) | File 7, no. 82 | Met | Met | Not met | Not included |
| Magde (2009) | File 6, no. 31 | Met | Not met | Not met | Not included |
| Maglunog et al. (2019) | File 1, no. 32 | Met | Not met | Met | Not included |
| Magolis et al. (2016) | File 5, no. 2 | Met | Not met | Not met | Not included |
| Mahdiuon et al. (2020) | File 2, no. 17 | Met | Not met | Met | Not included |
| Mahmood et al. (2018) | File 2, no. 18 | Met | Not met | Met | Not included |
| Maloney et al. (2014) | File 8, no. 64 | Met | Not met | Not met | Not included |
| Manca et al. (2017) | File 4, no. 56 | Met | Not met | Not met | Not included |
| Mandavgane (2016) | File 3, no. 92 | Met | Met | Not met | Not included |
| Mandviwalla et al. (2013) | File 8, no. 36 | Met | Met | Not met | Not included |
| Manlow et al. (2010) | File 9, no. 114 | Not met | Not met | Not met | Not included |
| Manson et al. (2018) | File 6, no. 25 | Not met | Not met | Not met | Not included |
| Manuel et al (2016) | File 3, no. 59 | Not met | Met | Not met | Not included |
| Marek et al. (2017) | File 6, no. 57 | Not met | Not met | Not met | Not included |
| Maresova et al. (2020) | File 1, no. 66 | Not met | Not met | Met | Not included |
| Marie Condie et al. (2018) | File 6, no. 53 | Met | Not met | Not met | Not included |
| Marshalsey et al. (2020) | File 3, no. 14 | Met | Met | Not met | Not included |
| Marsilio (2017) | File 4, no. 69 | Met | Not met | Not met | Not included |
| Martinez-Alemán (2014) | File 8, no. 61 | Not met | Not met | Not met | Not included |
| Martinez-Arbelaiz et al. (2017) | File 7, no. 7 | Met | Not met | Not met | Not included |
| Martinez-Cardama et al. (2019) | File 4, no. 60 | Met | Met | Met | Included |
| Marzo et al. (2017) | File 3, no. 63 | Met | Not met | Not met | Not included |
| Mastrodicasa et al. (2013) | File 8, no. 87 | Not met | Not met | Not met | Not included |
| Mazer et al. (2007) | File 6, no. 40 | Met | Not met | Not met | Not included |
| Mazer et al. (2009) | File 6, no. 79 | Met | Not met | Not met | Not included |
| Mazer et al. (2017) | File 7, no. 71 | Not met | Not met | Not met | Not included |
| McCarthy (2015) | File 4, no. 52 | Met | Met | Not met | Not included |
| McCarthy et al. (2014) | File 6, no. 69 | Met | Not met | Not met | Not included |
| McCorkle et al. (2012) | File 8, no. 96 | Met | Met | Not met | Not included |
| Mccorkle et al. (2017) | File 6, no. 96 | Met | Met | Not met | Not included |
| McEachern (2011) | File 5, no. 26 | Not met | Not met | Not met | Not included |
| McHaney et al. (2015) | File 8, no. 81 | Not met | Not met | Not met | Not included |
| Mclain (2019) | File 2, no. 62 | Met | Met | Met | Included |
| McNeill (2012) | File 8, no. 17 | Not met | Not met | Not met | Not included |
| Megele (2015) | File 8, no. 18 | Met | Met | Not met | Not included |
| Meishar-Tal et al. (2012) | File 3, no. 34 | Met | Met | Not met | Not included |
| Melchiorre et al. (2017) | File 7, no. 34 | Not met | Not met | Not met | Not included |
| Melton et al. (2011) | File 8, no. 30 | Not met | Met | Not met | Not included |
| Melton et al. (2018) | File 2, no. 89 | Met | Not met | Met | Not included |
| Mendez et al. (2014) | File 2, no. 42 | Not met | Not met | Not met | Not included |
| Menevse (2019) | File 2, no. 36 | Met | Not met | Met | Not included |
| Menzies et al. (2017) | File 3, no. 1 | Met | Not met | Not met | Not included |
| Meredith (2012) | File 8, no. 72 | Not met | Not met | Not met | Not included |
| Messner et al. (2016) | File 7, no. 38 | Not met | Met | Not met | Not included |
| Metzger (2015) | File 5, no. 17 | Not met | Not met | Not met | Not included |
| Mikum et al. (2018) | File 6, no. 72 | Met | Met | Not met | Not included |
| Milburn et al. (2014) | File 4, no. 49 | Met | Met | Not met | Not included |
| Miller (2013) | File 4, no. 47 | Met | Met | Met | Included |
| Miller (2017) | File 6, no. 50 | Met | Not met | Not met | Not included |
| Miller et al. (2007) | File 6, no. 20 | Not met | Not met | Not met | Not included |
| Mirembe et al. (2019) | File 2, no. 47 | Met | Not met | Met | Not included |
| Miron et al. (2015) | File 4, no. 37 | Met | Met | Not met | Not included |
| Mitchell (2012) | File 5, no. 3 | Met | Not met | Not met | Not included |
| Mnkandla et al. (2017) | File 4, no. 87 | Not met | Not met | Not met | Not included |
| Moghavvemi et al. (2018) | File 2, no. 40 | Met | Met | Met | Included |
| Molina et al. (2015) | File 8, no. 24 | Not met | Not met | Not met | Not included |
| Moll et al. (2017) | File 7, no. 22 | Met | Not met | Not met | Not included |
| Mondahl et al. (2014) | File 6, no. 67 | Met | Met | Not met | Not included |
| Monseau et al. (2017) | File 6, no. 43 | Not met | Not met | Not met | Not included |
| Montoneri (2015) | File 2, no. 39 | Met | Met | Met | Included |
| Montoneri (2017) | File 1, no. 30 | Met | Met | Met | Included |
| Moore (2009) | File 9, no. 121 | Not met | Not met | Not met | Not included |
| Moore (2012) | File 5, no. 30 | Not met | Not met | Not met | Not included |
| Moreno et al. (2012) | File 5, no. 12 | Met | Not met | Not met | Not included |
| Morris et al. (2009) | File 6, no. 30 | Met | Not met | Not met | Not included |
| Mostafa (2015) | File 7, no. 29 | Met | Not met | Not met | Not included |
| Muhametjanova et al. (2019) | File 4, no. 70 | Met | Not met | Not met | Not included |
| Munoz et al. (2014) | File 6, no. 92 | Met | Met | Not met | Not included |
| Muñoz et al. (2015) | File 7, no. 93 | Not met | Not met | Not met | Not included |
| Murray et al. (2019) | File 5, no. 10 | Met | Not met | Not met | Not included |
| Murugaiah et al. (2019) | File 4, no. 55 | Met | Met | Not met | Not included |
| Nadelson et al. (2017) | File 4, no. 58 | Met | Not met | Not met | Not included |
| Nagel et al. (2018) | File 3, no. 40 | Met | Not met | Met | Not included |
| Naghdipour et al. (2016) | File 3, no. 47 | Met | Met | Met | Included |
| Nairn et al. (2018) | File 2, no. 35 | Met | Not met | Not met | Not included |
| Nantwi et al. (2017) | File 7, no. 69 | Not met | Not met | Not met | Not included |
| Narayan et al. (2019) | File 4, no. 21 | Met | Met | Met | Included |
| Nathan et al. (2014) | File 6, no. 39 | Met | Met | Not met | Not included |
| Nazir et al. (2019) | File 1, no. 11 | Met | Met | Met | Included |
| Nehls (2014) | File 4, no. 81 | Met | Not met | Not met | Not included |
| Neier et al. (2015) | File 7, no. 80 | Met | Not met | Not met | Not included |
| Nekritz (2011) | File 8, no. 40 | Not met | Not met | Not met | Not included |
| Neville et al. (2013) | File 6, no. 95 | Met | Met | Not met | Not included |
| Nkhoma et al. (2015) | File 4, no. 36 | Met | Met | Met | Included |
| Norman et al. (2015) | File 5, no. 29 | Met | Not met | Not met | Not included |
| Normore et al. (2007) | File 9, no. 124 | Not met | Not met | Not met | Not included |
| Nwangwa et al. (2014) | File 6, no. 87 | Met | Not met | Not met | Not included |
| O'Bannon et al. (2014) | File 4, no. 89 | Met | Met | Met | Included |
| O'Brien et al. (2018) | File 3, no. 51 | Met | Met | Not met | Not included |
| Odewumi et al. (2018) | File 3, no. 99 | Met | Not met | Not met | Not included |
| Odom et al. (2013) | File 8, no. 65 | Met | Not met | Not met | Not included |
| Odom et al. (2019) | File 4, no. 95 | Met | Met | Not met | Not included |
| Oelze (2019) | File 4, no. 44 | Not met | Not met | Not met | Not included |
| Oh et al. (2014) | File 5, no. 13 | Met | Not met | Not met | Not included |
| Okoro (2012) | File 8, no. 32 | Not met | Not met | Not met | Not included |
| Okoroma et al. (2018) | File 3, no. 84 | Met | Not met | Not met | Not included |
| Olson et al. (2009) | File 6, no. 58 | Met | Not met | Not met | Not included |
| Olutola et al. (2016) | File 5, no. 9 | Met | Not met | Not met | Not included |
| Omoera et al. (2018) | File 4, no. 45 | Met | Not met | Not met | Not included |
| Oravec (2012) | File 7, no. 11 | Not met | Not met | Not met | Not included |
| Orawiwatnakul et al. (2016) | File 2, no. 4 | Met | Met | Met | Included |
| Oregon et al. (2019) | File 3, no. 18 | Not met | Not met | Not met | Not included |
| Owan et al. (2020) | File 1, no. 88 | Met | Not met | Met | Not included |
| Owens et al. (2017) | File 1, no. 86 | Met | Met | Met | Included |
| Ozdemir. 2017 | File 3, no. 64 | Met | Met | Not met | Not included |
| Pai et al. (2017) | File 2, no. 10 | Met | Met | Met | Included |
| Pai et al. (2017) | File 2, no. 93 | Met | Met | Met | Included |
| Palen (2008) | File 9, no. 116 | Not met | Not met | Not met | Not included |
| Paris et al. (2015) | File 2, no. 28 | Met | Met | Not met | Not included |
| Parrot et al. (2010) | File 9, no. 125 | Met | Not met | Not met | Not included |
| Pasquini et al. (2017) | File 6, no. 68 | Not met | Not met | Not met | Not included |
| Pavlik et al. (2015) | File 5, no. 38 | Not met | Not met | Not met | Not included |
| Pearson (2010) | File 9, no. 118 | Not met | Not met | Not met | Not included |
| Peeters et al. (2020) | File 1, no. 29 | Met | Met | Met | Included |
| Peluchette et al. (2010) | File 6, no. 27 | Met | Not met | Not met | Not included |
| Pempek et al. (2009) | File 6, no. 18 | Met | Not met | Not met | Not included |
| Persson et all. (2016) | File 7, no. 45 | Not met | Not met | Not met | Not included |
| Peruta et al. (2017) | File 1, no. 64 | Not met | Not met | Met | Not included |
| Peruta et al. (2018) | File 1, no. 47 | Not met | Not met | Met | Not included |
| Petrovic et al. (2014) | File 4, no. 39 | Met | Met | Not met | Not included |
| Pham (2014) | File 8, no. 43 | Not met | Not met | Not met | Not included |
| Phillips (2012) | File 5, no. 4 | Not met | Not met | Not met | Not included |
| Pickering et al. (2017) | File 3, no. 58 | Met | Not met | Met | Not included |
| Pikalek (2010) | File 8, no. 44 | Not met | Not met | Not met | Not included |
| Pimmer et al. (2012) | File 5, no. 31 | Met | Not met | Not met | Not included |
| Pimmer et al. (2017) | File 1, no. 26 | Not met | Met | Not met | Not included |
| Ping et al. (2015) | File 2, no. 76 | Met | Met | Met | Included |
| Piotrowski (2015a) | File 5, no. 22 | Not met | Not met | Not met | Not included |
| Piotrowski (2015b) | File 7, no. 53 | Not met | Not met | Not met | Not included |
| Piotrowski (2015c) | File 7, no. 59 | Not met | Not met | Not met | Not included |
| Pitiporntapin et al. (2015) | File 7, no. 97 | Met | Met | Not met | Not included |
| Platts (2019) | File 4, no. 12 | Met | Not met | Not met | Not included |
| Poelhuber et al. (2011) | File 7, no. 25 | Met | Not met | Not met | Not included |
| Polsgrove et al. (2013) | File 8, no. 2 | Met | Not met | Not met | Not included |
| Popescu et al. (2020) | File 2, no. 23 | Met | Met | Met | Included |
| Poth et al. (2016) | File 3, no. 76 | Met | Not met | Not met | Not included |
| Powers et al. (2012) | File 8, no. 49 | Not met | Not met | Not met | Not included |
| Premadasa (2019) | File 1, no. 55 | Met | Not met | Met | Not included |
| Prescott (2014) | File 4, no. 74 | Not met | Not met | Not met | Not included |
| Prescott et al. (2013) | File 5, no. 35 | Met | Not met | Not met | Not included |
| Pringle et al. (2019) | File 4, no. 83 | Not met | Not met | Not met | Not included |
| Purnamasari (2019) | File 1, no. 52 | Met | Met | Met | Included |
| Qi (2019) | File 4, no. 64 | Met | Not met | Not met | Not included |
| Quadri et al. (2016) | File 7, no. 73 | Not met | Not met | Not met | Not included |
| Quan-Haase et al. (2010) | File 1, no. 91 | Met | Not met | Met | Not included |
| Rahman et al. (2020) | File 2, no. 38 | Met | Met | Met | Included |
| Ramadan (2017) | File 3, no. 91 | Met | Not met | Not met | Not included |
| Ramazanoglu et al. (2018) | File 1, no. 74 | Met | Not met | Met | Not included |
| Rambe (2012a) | File 5, no. 5 | Met | Met | Not met | Not included |
| Rambe (2012b) | File 5, no. 16 | Met | Met | Not met | Not included |
| Rambe (2012c) | File 7, no. 16 | Met | Met | Not met | Not included |
| Rambe (2013) | File 1, no. 12 | Met | Not met | Not met | Not included |
| Rambe et al. (2014) | File 4, no. 53 | Met | Not met | Not met | Not included |
| Rambe et al. (2015) | File 7, no. 83 | Not met | Not met | Not met | Not included |
| Ramdani et al. (2020) | File 1, no. 72 | Met | Met | Not met | Not included |
| Ramspott (2013) | File 8, no. 84 | Not met | Not met | Not met | Not included |
| Raza et al. (2017) | File 3, no. 35 | Met | Not met | Not met | Not included |
| Razak et al. (2015) | File 2, no. 19 | Met | Met | Not met | Not included |
| Reich (2010) | File 6, no. 8 | Met | Not met | Not met | Not included |
| Remund (2015) | File 7, no. 43 | Met | Met | Not met | Not included |
| Reynolds et al. (2017) | File 6, no. 61 | Not met | Not met | Not met | Not included |
| Rheingold (2008) | File 9, no. 126 | Not met | Not met | Not met | Not included |
| Rheingold (2010) | File 9, no. 101 | Not met | Not met | Not met | Not included |
| Rhodes et al. (2015) | File 2, no. 43 | Met | Not met | Met | Not included |
| Riady (2014) | File 2, no. 11 | Met | Met | Met | Included |
| Richman et al. (2017) | File 3, no. 36 | Met | Not met | Not met | Not included |
| Riley (2015) | File 2, no. 14 | Met | Not met | Not met | Not included |
| Riser et al. (2020) | File 2, no. 57 | Met | Not met | Met | Not included |
| Roberson et al. (2018) | File 6, no. 52 | Met | Not met | Not met | Not included |
| Roblyer et al. (2010) | File 6, no. 36 | Met | Not met | Not met | Not included |
| Rodliyah (2016) | File 3, no. 93 | Met | Met | Not met | Not included |
| Roebuck et al. (2013) | File 8, no. 22 | Not met | Not met | Not met | Not included |
| Romero-Hall (2017) | File 6, no. 56 | Met | Not met | Not met | Not included |
| Romero-Hall (2018) | File 6, no. 65 | Not met | Not met | Not met | Not included |
| Root et al. (2014) | File 8, no. 73 | Met | Not met | Not met | Not included |
| Rosatelli (2015) | File 7, no. 12 | Not met | Not met | Not met | Not included |
| Rosenberg et al. (2016) | File 7, no. 19 | Not met | Met | Not met | Not included |
| Rosli et al. (2016) | File 5, no. 21 | Met | Not met | Not met | Not included |
| Rotboim et al. (2019) | File 1, no. 50 | Met | Not met | Met | Not included |
| Rouis et al. (2011) | File 6, no. 41 | Met | Not met | Not met | Not included |
| Rowe (2014) | File 8, no. 74 | Met | Not met | Not met | Not included |
| Rubrico et al. (2014) | File 4, no. 41 | Met | Met | Met | Included |
| Rudick et al. (2019) | File 4, no. 100 | Not met | Not met | Not met | Not included |
| Russell (2013) | File 6, no. 54 | Not met | Not met | Not met | Not included |
| Russell et al. (2012) | File 5, no. 8 | Not met | Not met | Not met | Not included |
| Rustan (2020) | File 1, no. 10 | Met | Not met | Met | Not included |
| Sacks et al. (2012) | File 8, no. 27 | Not met | Not met | Not met | Not included |
| Sahin (2017) | File 4, no. 80 | Met | Not met | Not met | Not included |
| Saifudin et al. (2016) | File 2, no. 77 | Met | Met | Met | Included |
| Saini et al. (2019) | File 4, no. 54 | Met | Not met | Not met | Not included |
| Salameh (2017) | File 1, no. 6 | Met | Met | Met | Included |
| Salmon et al. (2015) | File 8, no. 91 | Met | Met | Not met | Not included |
| Samuel-Peretz et al. (2017) | File 7, no. 24 | Met | Met | Not met | Not included |
| Samuels (2011) | File 8, no. 21 | Not met | Not met | Not met | Not included |
| Sanderson (2018) | File 6, no. 84 | Not met | Not met | Not met | Not included |
| Sandlin et al. (2014) | File 8, no. 8 | Met | Not met | Not met | Not included |
| Santos (2011) | File 9, no. 112 | Not met | Not met | Not met | Not included |
| Santoveña-Casal (2019) | File 2, no. 79 | Met | Not met | Met | Not included |
| Sapsani (2019) | File 1, no. 33 | Met | Not met | Met | Not included |
| Schirr (2013) | File 8, no. 12 | Not met | Not met | Not met | Not included |
| Schmeichel et al. (2018) | File 4, no. 76 | Met | Not met | Not met | Not included |
| Schoper et al. (2017) | File 1, no. 97 | Met | Met | Not met | Not included |
| Schroeder et al. (2009) | File 6, no. 78 | Met | Met | Met | Included |
| Sciutto (2015) | File 4, no. 93 | Met | Not met | Not met | Not included |
| Seechalio (2014) | File 8, no. 39 | Not met | Not met | Not met | Not included |
| Selwyn (2009) | File 6, no. 34 | Met | Not met | Not met | Not included |
| Sessa (2015) | File 8, no. 78 | Not met | Not met | Not met | Not included |
| Setiawan et al. (2020) | File 1, no. 77 | Met | Not met | Met | Not included |
| Settle et al. (2012) | File 8, no. 63 | Met | Not met | Not met | Not included |
| Shafie et al. (2016a) | File 2, no. 25 | Met | Met | Not met | Not included |
| Shafie et al. (2016b) | File 2, no. 52 | Met | Met | Not met | Not included |
| Shafie et al. (2016c) | File 2, no. 55 | Met | Met | Not met | Not included |
| Sharma et al. (2017) | File 6, no. 64 | Not met | Not met | Not met | Not included |
| Shaw (2016) | File 3, no. 19 | Met | Not met | Not met | Not included |
| Sheeran et al. (2018) | File 2, no. 7 | Met | Met | Met | Included |
| Sheffield et al. (2016) | File 2, no. 48 | Not met | Met | Not met | Not included |
| Shelton (2017) | File 7, no. 37 | Not met | Not met | Not met | Not included |
| Shen (2019) | File 4, no. 68 | Met | Not met | Not met | Not included |
| Shensa et al. (2018) | File 6, no. 59 | Met | Not met | Not met | Not included |
| Shephard et al. (2019) | File 4, no. 34 | Met | Not met | Not met | Not included |
| Sherrell et al. (2016) | File 3, no. 5 | Met | Met | Not met | Not included |
| Shields et al. (2019) | File 4, no. 61 | Met | Not met | Not met | Not included |
| Shih (2011) | File 6, no. 13 | Met | Met | Met | Included |
| Shih (2013) | File 3, no. 24 | Met | Met | Not met | Not included |
| Shorkey et al. (2014) | File 8, no. 25 | Not met | Met | Not met | Not included |
| Simiyu et al. (2020) | File 3, no. 71 | Met | Not met | Not met | Not included |
| Simons et al. (2016) | File 7, no. 81 | Not met | Not met | Not met | Not included |
| Simpson (2012) | File 3, no. 29 | Not met | Met | Not met | Not included |
| Simsek et al. (2014) | File 6, no. 81 | Met | Not met | Not met | Not included |
| Simsek et al. (2019) | File 2, no. 2 | Met | Not met | Met | Not included |
| Singh (2013) | File 3, no. 42 | Met | Met | Not met | Not included |
| Singh (2017) | File 6, no. 48 | Met | Not met | Not met | Not included |
| Sittiwong et al. (2015) | File 2, no. 75 | Met | Met | Met | Included |
| Skerrett (2010) | File 6, no. 45 | Met | Met | Not met | Not included |
| Sleeman et al. (2016) | File 7, no. 66 | Not met | Not met | Not met | Not included |
| Slim et al. (2019) | File 2, no. 61 | Met | Met | Met | Included |
| Smith (2014) | File 8, no. 37 | Met | Met | Not met | Not included |
| Snow (2017) | File 6, no. 63 | Not met | Not met | Not met | Not included |
| So et al. (2013) | File 8, no. 53 | Met | Not met | Not met | Not included |
| Sobieraj et al., 2001 | File 9, no. 109 | Not met | Not met | Not met | Not included |
| Sofowora (2013) | File 6, no. 83 | Met | Not met | Not met | Not included |
| Solmaz (2017) | File 7, no. 2 | Met | Not met | Not met | Not included |
| Solmaz (2018) | File 1, no. 48 | Met | Not met | Met | Not included |
| Song (2017) | File 2, no. 69 | Met | Met | Not met | Not included |
| Sorensen-Unruh (2017) | File 7, no. 15 | Met | Met | Not met | Not included |
| Spackman et al. (2017) | File 6, no. 26 | Not met | Not met | Not met | Not included |
| Spikes et al. (2014) | File 8, no. 1 | Met | Met | Not met | Not included |
| Stander et al. (2017) | File 7, no. 51 | Met | Not met | Not met | Not included |
| Steinbrecher et al. (2012) | File 5, no. 25 | Met | Not met | Not met | Not included |
| Stephen (2014) | File 2, no. 49 | Not met | Not met | Not met | Not included |
| Stephenson-Abetz et al. (2012) | File 5, no. 40 | Met | Not met | Not met | Not included |
| Stirling (2016) | File 3, no. 80 | Met | Not met | Not met | Not included |
| Stoller (2013) | File 8, no. 47 | Not met | Not met | Not met | Not included |
| Strayhorn (2012) | File 5, no. 28 | Met | Not met | Not met | Not included |
| Sumida Garcia et al. (2017) | File 7, no. 23 | Met | Not met | Not met | Not included |
| Sutherland et al. (2019) | File 2, no. 12 | Not met | Not met | Met | Not included |
| Sutherland et al. (2020) | File 1, no. 2 | Not met | Not met | Met | Not included |
| Suwinyattichaiporn (2016) | File 7, no. 55 | Not met | Met | Not met | Not included |
| Syme (2013) | File 8, no. 77 | Not met | Not met | Not met | Not included |
| Szeto et al. (2016) | File 7, no. 44 | Met | Not met | Not met | Not included |
| Tananuraksakul (2014) | File 2, no. 90 | Met | Met | Not met | Not included |
| Tananuraksakul (2015) | File 2, no. 9 | Met | Met | Not met | Not included |
| Tay et al. (2011) | File 8, no. 14 | Not met | Met | Not met | Not included |
| Taylor (2019) | File 4, no. 99 | Not met | Not met | Not met | Not included |
| Taylor et al. (2012) | File 3, no. 37 | Met | Not met | Not met | Not included |
| Tayo et al. (2019) | File 2, no. 63 | Met | Not met | Met | Not included |
| Teclehaimanot et al. (2011) | File 6, no. 74 | Met | Not met | Not met | Not included |
| Teixeira et al. (2017) | File 6, no. 76 | Met | Met | Met | Included |
| Tenorio de Azevedo (2019) | File 4, no. 1 | Not met | Not met | Not met | Not included |
| Teo (2016) | File 3, no. 61 | Met | Not met | Not met | Not included |
| Teo et al. (2018) | File 2, no. 84 | Met | Not met | Met | Not included |
| Teruya et al. (2014) | File 8, no. 59 | Not met | Not met | Not met | Not included |
| Thompson et al. (2012) | File 5, no. 37 | Met | Not met | Not met | Not included |
| Tiggemann et al. (2010) | File 9, no. 123 | Met | Not met | Not met | Not included |
| Torun (2020) | File 1, no. 17 | Met | Not met | Met | Not included |
| Tran (2016) | File 2, no. 87 | Met | Met | Met | Included |
| Tras et al. (2019) | File 1, no. 56 | Met | Not met | Met | Not included |
| Tu (2018) | File 3, no. 28 | Met | Not met | Not met | Not included |
| Tucker (2015) | File 7, no. 95 | Met | Met | Met | Included |
| Tugrul (2017) | File 1, no. 49 | Met | Met | Met | Included |
| Tull et al. (2017) | File 4, no. 59 | Not met | Not met | Not met | Not included |
| Tur et al. (2015) | File 5, no. 24 | Met | Met | Met | Included |
| Tuten et al. (2012) | File 8, no. 83 | Not met | Not met | Not met | Not included |
| Tutgun-Unal (2020) | File 1, no. 58 | Met | Not met | Met | Not included |
| Tynes et al. (2013) | File 8, no. 20 | Met | Not met | Not met | Not included |
| Unsal (2018) | File 4, no. 86 | Met | Not met | Not met | Not included |
| Vaccaro et al. (2015) | File 7, no. 89 | Met | Not met | Not met | Not included |
| Valiente-Neighbours (2020) | File 3, no. 43 | Not met | Met | Not met | Not included |
| Valjataga et al. (2009) | File 9, no. 120 | Met | Met | Not met | Not included |
| van Beynen et al. (2016) | File 3, no. 33 | Not met | Not met | Met | Not included |
| Van Den Beemt et al. (2020) | File 3, no. 89 | Not met | Not met | Not met | Not included |
| van Rooyen et al. (2020) | File 3, no. 75 | Met | Not met | Not met | Not included |
| VanDoorn et al. (2013) | File 1, no. 20 | Met | Met | Met | Included |
| Veeck et al. (2014) | File 8, no. 92 | Met | Met | Not met | Not included |
| Veletsianos (2013) | File 8, no. 46 | Not met | Not met | Not met | Not included |
| Veletsianos et al. (2019) | File 4, no. 8 | Not met | Not met | Not met | Not included |
| Vie (2015) | File 7, no. 98 | Not met | Not met | Not met | Not included |
| Vinuales et al. (2017) | File 7, no. 6 | Met | Not met | Not met | Not included |
| Vivakaran et al. (2018) | File 3, no. 82 | Not met | Not met | Not met | Not included |
| Vivakaran et al. (2018) | File 6, no. 98 | Not met | Not met | Not met | Not included |
| Vivian et al. (2014) | File 4, no. 75 | Met | Not met | Not met | Not included |
| Voivonta et al. (2018) | File 2, no. 33 | Not met | Not met | Not met | Not included |
| Vrontis et al. (2018) | File 6, no. 80 | Met | Not met | Not met | Not included |
| Wandera et al. (2016) | File 7, no. 86 | Met | Not met | Not met | Not included |
| Wang et al. (2013) | File 3, no. 55 | Met | Met | Met | Included |
| Wang et al. (2014a) | File 4, no. 72 | Met | Not met | Not met | Not included |
| Wang et al. (2014b) | File 8, no. 85 | Met | Met | Not met | Not included |
| Wanjohi et al. (2015) | File 2, no. 58 | Met | Not met | Met | Not included |
| Warner et al. (2017) | File 3, no. 21 | Not met | Not met | Not met | Not included |
| Watson (2017) | File 7, no. 46 | Not met | Not met | Not met | Not included |
| Weiqin et al. (2016) | File 3, no. 68 | Met | Not met | Not met | Not included |
| West (2017) | File 6, no. 19 | Met | Not met | Not met | Not included |
| Whittaker et al. (2014) | File 4, no. 30 | Met | Met | Met | Included |
| Whittaker et al. (2015) | File 7, no. 17 | Met | Not met | Not met | Not included |
| Wichadee (2013) | File 3, no. 62 | Met | Met | Not met | Not included |
| Wicks et al. (2020) | File 3, no. 9 | Not met | Not met | Not met | Not included |
| Willems et al. (2018) | File 6, no. 23 | Not met | Not met | Not met | Not included |
| Williams et al. (2017) | File 4, no. 90 | Met | Not met | Not met | Not included |
| Willoughby et al. (2017) | File 4, no. 28 | Met | Not met | Not met | Not included |
| Wilner et al. (2018) | File 2, no. 27 | Met | Not met | Met | Not included |
| Winkelmann et al. (2018) | File 6, no. 11 | Met | Not met | Not met | Not included |
| Witte (2014) | File 4, no. 17 | Not met | Not met | Not met | Not included |
| Woodley et al. (2012) | File 8, no. 76 | Met | Not met | Not met | Not included |
| Woodley et al. (2014) | File 8, no. 88 | Not met | Not met | Not met | Not included |
| Woods et al. (2019) | File 4, no. 94 | Met | Met | Not met | Not included |
| Wright et al. (2017) | File 7, no. 36 | Met | Not met | Not met | Not included |
| Wu et al. (2012) | File 3, no. 85 | Met | Not met | Not met | Not included |
| Wu et al. (2020) | File 1, no. 98 | Met | Met | Met | Included |
| Xu et al. (2014) | File 6, no. 1 | Met | Not met | Not met | Not included |
| Yagci (2015) | File 5, no. 11 | Met | Met | Met | Included |
| Yaman (2016) | File 2, no. 26 | Met | Not met | Met | Not included |
| Yaman (2020) | File 1, no. 44 | Met | Not met | Met | Not included |
| Yang (2018) | File 1, no. 60 | Met | Not met | Not met | Not included |
| Yang et al. (2020) | File 1, no. 42 | Met | Not met | Met | Not included |
| Yavich et al. (2019) | File 2, no. 59 | Met | Not met | Met | Not included |
| Yeh et al. (2019) | File 2, no. 60 | Met | Not met | Met | Not included |
| Yeh et al. (2020) | File 3, no. 16 | Met | Met | Not met | Not included |
| Yen et al. (2015) | File 4, no. 13 | Met | Met | Not met | Not included |
| Yen et al. (2015b) | File 8, no. 7 | Met | Not met | Not met | Not included |
| Yeo (2014) | File 6, no. 62 | Met | Not met | Not met | Not included |
| Yesilyurt et al. (2020) | File 1, no. 51 | Met | Not met | Met | Not included |
| Yildirim et al. (2018) | File 4, no. 50 | Met | Not met | Not met | Not included |
| Young (2009) | File 9, no. 111 | Not met | Not met | Not met | Not included |
| Young et al. (2014) | File 8, no. 67 | Not met | Not met | Not met | Not included |
| Yu (2014) | File 2, no. 1 | Met | Met | Met | Included |
| Zachos et al. (2018) | File 4, no. 65 | Not met | Not met | Not met | Not included |
| Zgheib et al. (2020) | File 1, no. 65 | Not met | Met | Met | Not included |
| Zhang et al. (2014) | File 4, no. 88 | Met | Met | Met | Included |
| Zhang et al. (2016) | File 4, no. 18 | Met | Met | Not met | Not included |
| Zhu (2019) | File 4, no. 62 | Met | Not met | Not met | Not included |
| Zhu et al. (2020) | File 3, no. 90 | Met | Not met | Not met | Not included |
| Zhuravleva et al. (2016) | File 7, no. 96 | Not met | Not met | Not met | Not included |
| Zickar et al. (2018) | File 2, no. 95 | Not met | Not met | Met | Not included |
| Zimmerman et al. (2020) | File 1, no. 28 | Met | Not met | Not met | Not included |

*Notes*.

Criterion 1, Undergraduate or graduate level students; Criterion 2, Use of social media for educational purposes; Criterion 3, Quantitative variable of engagement in social media.

Archival reference files can be downloaded from <https://www.dropbox.com/s/tus5zkwf8wom7r1/Archival%20Reference%20Files.zip?dl=0>
